# Supplementary material for: Evidence of health system resilience in Myanmar during Cyclone Nargis: a qualitative analysis
Source: BMJ Open. 2021 Sep 22;11(9):e050700. doi: 10.1136/bmjopen-2021-050700 (PMC8461277; doi:10.1136/bmjopen-2021-050700)
Supplement: Supplementary data [file bmjopen-2021-050700supp001.pdf]

SUPPLEMENTARY FILE. INTERVIEW GUIDE

Study Title: Health System Resilience: key for unlocking the complex health system strengthening agenda

Investigator: ______________________ **Phone number:**_________________________

Organisation/Department: ____________________________ **Date:** |__|__|__|__|__|__|__|__|

Position/Title: ____

Stakeholder Group:

□ Central Government

□ Local Government (Township, local health committees, member of parliament, etc)

□ Health workers

□ Int'l Agencies (UN, NGOs, Bilateral, etc)

Number of participants: |__|__|

DRAFT SEMI-STRUCTURED IN-DEPTH INTERVIEW QUESTIONS

Introduction: purpose of research, use of audio tape recorder, confidentiality

I. PARTICIPANT INTRODUCTION

1. What is your role when the country is hit with an external shock, whether it be a natural disaster, unforeseen pandemic, etc.

    Prompt: examples of disaster management, pandemic, natural disasters

II. CASE 1: CYCLONE NARGIS (2008)

2. (General) Can you please describe in detail the account of Cyclone Nargis?

    Probe: What was your title/position at the time?

    What was the response protocol (written or perceived norm)?

    What actions (out of the norm) did you take?

    How did you respond as an individual?

3. (General) Can you please describe in detail the response actions taken as an organisation that you were a part of at the time?

    Probe: Decision-making protocol? Evidence?

4. (General) Can you comment on one strength and weakness that the health system exhibited during Cyclone Nargis?

Probe: Which key element was present or missing during the incident?

5. (Dimension 1_Aware) At the time of Cyclone Nargis, how was the health system monitoring and tracking its health risks?

6. (Dimension 1_Aware) Once the disaster hit, who was included in the communication channel?

7. (Dimension 1_Aware) Who made the decisions regarding resource allocation and coordination efforts?

8. (Dimension 2_Diverse) At the time of Cyclone Nargis, were a range of health services still offered in health facilities (business as usual) or was the quality/quantity of services compromised? Why?

9. (Dimension 3_Self-regulating) Can you recall the extent of non-state organisations intervening to provide support at the time of crisis? (e.g. NGOs, Int'l Orgnisations, private actors, etc)

10. (Dimension 3_Self-regulating) At national level, were there reserve capacity (budget, HR, other resources) to summon quickly?

11. (Dimension 4_Integrated) Can you comment on the level of coordination between government, global and private actors at the time of the crisis?

12. (Dimension 4_Integrated) Who was the main actor responsible for coordination?

13. (Dimension 5_Adaptive) Was there at the time flexibility when it came to spending budget to respond to the emergency?

14. (Dimension 5_Adaptive) Can you comment on how much decision-making was possible at the decentralised/local level?

15. What was the main lessons learnt after undergoing Cyclone Nargis?

III. CONCEPT OF HEALTH SYSTEM RESILIENCE (HSR) AND GENERAL QUESTIONS

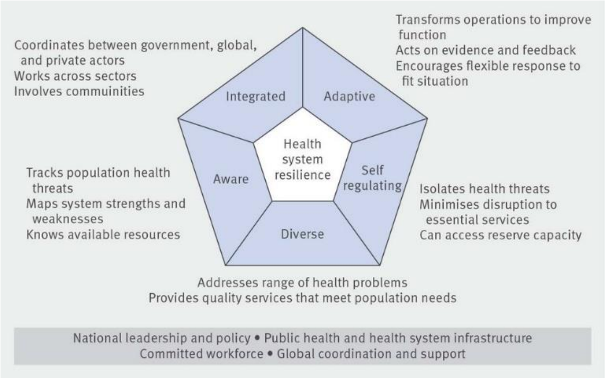

Figure 1. Resilient Health System Framework

16. Reviewing the framework above, how would you describe Myanmar's health system resilience generally?

Probe: Can you give concrete examples that indicate Myanmar's HSR?

17. What is Myanmar's strength/weakness when it comes to resilience?

18. What do you think is the most important element of resilience?

IV. PREPAREDNESS AND FUTURE SHOCKS

19. What may be the biggest threat/risk for Myanmar?

20. At this point in time, how would you describe Myanmar's preparedness for future shocks?

21. What national policies are in place to enable the Ministry of Health to be resilient to future shocks?

22. Can you comment on the efforts taken by the Ministry of Health and Sports to improve preparedness? (HR allocation, crisis management team, emergency plan, trainings undertaken, protocols, etc)

23. What would you advise to improve health system resilience in Myanmar?

Thank you for your time and heartfelt responses to the interview questions.
