# Supplementary material for: Evidence of health system resilience in Myanmar during Cyclone Nargis: a qualitative analysis
Source: BMJ Open. 2021 Sep 22;11(9):e050700. doi: 10.1136/bmjopen-2021-050700 (PMC8461277; doi:10.1136/bmjopen-2021-050700)
Supplement: Author's manuscript [file bmjopen-2021-050700.draft_revisions.pdf]

BMJ Open is committed to open peer review. As part of this commitment we make the peer review history of every article we publish publicly available.

When an article is published we post the peer reviewers' comments and the authors' responses online. We also post the versions of the paper that were used during peer review. These are the versions that the peer review comments apply to.

The versions of the paper that follow are the versions that were submitted during the peer review process. They are not the versions of record or the final published versions. They should not be cited or distributed as the published version of this manuscript.

BMJ Open is an open access journal and the full, final, typeset and author-corrected version of record of the manuscript is available on our site with no access controls, subscription charges or pay-per-view fees (http://bmjopen.bmj.com).

If you have any questions on BMJ Open's open peer review process please email info.bmjopen@bmj.com

BMJ Open

Evidence of health system resilience in Myanmar during Cyclone Nargis: perspectives from international organisations

Journal:	*BMJ Open*
Manuscript ID	bmjopen-2021-050700
Article Type:	Original research
Date Submitted by the Author:	02-Mar-2021

Complete List of Authors:	Grimm, Pauline Yongeun; Swiss Tropical and Public Health Institute; University of Basel<br>Merten, Sonja; Swiss Tropical and Public Health Institute; University of Basel<br>Wyss, Kaspar; Swiss Tropical and Public Health Institute; University of Basel

Keywords:	QUALITATIVE RESEARCH, PUBLIC HEALTH, Health policy < HEALTH SERVICES ADMINISTRATION & MANAGEMENT

SCHOLARONE™
Manuscripts

I, the Submitting Author has the right to grant and does grant on behalf of all authors of the Work (as defined in the below author licence), an exclusive licence and/or a non-exclusive licence for contributions from authors who are: i) UK Crown employees; ii) where BMJ has agreed a CC-BY licence shall apply, and/or iii) in accordance with the terms applicable for US Federal Government officers or employees acting as part of their official duties; on a worldwide, perpetual, irrevocable, royalty-free basis to BMJ Publishing Group Ltd ("BMJ") its licensees and where the relevant Journal is co-owned by BMJ to the co-owners of the Journal, to publish the Work in this journal and any other BMJ products and to exploit all rights, as set out in our licence.

The Submitting Author accepts and understands that any supply made under these terms is made by BMJ to the Submitting Author unless you are acting as an employee on behalf of your employer or a postgraduate student of an affiliated institution which is paying any applicable article publishing charge ("APC") for Open Access articles. Where the Submitting Author wishes to make the Work available on an Open Access basis (and intends to pay the relevant APC), the terms of reuse of such Open Access shall be governed by a Creative Commons licence – details of these licences and which Creative Commons licence will apply to this Work are set out in our licence referred to above.

Other than as permitted in any relevant BMJ Author's Self Archiving Policies, I confirm this Work has not been accepted for publication elsewhere, is not being considered for publication elsewhere and does not duplicate material already published. I confirm all authors consent to publication of this Work and authorise the granting of this licence.

For peer review only

Evidence of health system resilience in Myanmar during Cyclone Nargis: perspectives from international organisations

Pauline Yongeun Grimm*, Sonja Merten, Kaspar Wyss

***corresponding author: Mühlegasse 49a, 4314 Zeiningen, Switzerland / pauline.grimm@unibas.ch**

Affiliations for all authors:

1. **Swiss Tropical and Public Health Institute, Basel, Switzerland**
2. **University of Basel, Basel, Switzerland**

ABSTRACT

Objective

The aim of this study is to improve the understanding of the characteristics of health system resilience in Myanmar's response to Cyclone Nargis and to explore ways to improve resilience at the system level.

Design and Setting

This is an explanatory qualitative study exploring the institutional capacity of resilience in Myanmar's health system. Analysis proceeded using a data-driven thematic analysis closely following the framework method. This process enabled comparisons and contrasts of key emergent themes between the participants, which later generated key results describing the foundational assets, barriers and opportunities for achieving resilience in Myanmar.

Participants

The study comprised of 12 in-depth interviews conducted with representatives from international organisations and non-governmental organisations. The inclusion criteria to recruiting the participants were that they had directly been a part of the Cyclone Nargis response at the time. There was a balanced distribution of participants across UN, bilateral, and NGOs and most of them were either Myanmar citizens or expatriates with extensive working experience based in Myanmar.

Results

Key findings elucidate the characteristics of resilience that have been salient or absent in Myanmar's response to Cyclone Nargis. Strong social capital and motivation propelled by its deep-rooted culture and religion served as Myanmar's greatest assets that filled major gaps in the system. Meanwhile, its post-colonial and military legacy posed barriers towards investing in building its long-term foundations towards resilience.

Conclusions

This study revealed that resilience in the health system can be built through strategic investments towards building the foundations of resilience to better prepare for future shocks. In the case of Myanmar, social capital and motivation, which surfaced as its foundational assets can be channelled into opportunities that can help achieve its long-term health goals, accelerating its journey towards resilience in the health system.

Strengths and limitations of this study

- This study tests the applicability of a conceptual framework for the first time in a low-income country and builds on the health system resilience conceptual framework developed from a previous systematic review.

For peer review only - http://bmjopen.bmj.com/site/about/guidelines.xhtml
For peer review only

- The topic of health system resilience has a potential to make a timely contribution to the health systems and policy research community, with lessons generated that can be transferred to other contexts.
- The study only represents the view of international organisations and non-governmental organisations, restricting the scope of study to the perceptions of a single stakeholder group, which limits the generalizability of the findings.

INTRODUCTION

Health system resilience

The 2014-15 Ebola epidemic in West Africa served as a turning point in global health. Resilience was introduced as a concept relevant in the discourses for health system strengthening as well as for crisis management in fragile settings (1). Since then, there has been a plethora of literature trying to define, measure, and characterize the concept (2), (3), (4). Resilience is once again receiving more attention than ever with the failure to contain the on-going battle against Covid-19 pandemic.

Health system resilience is defined as the capacity to prepare for and effectively respond to crises whilst retaining core health system functions when a crisis hits. An important aspect concerns the capability of the system to reorganise itself to meet the evolving needs of the situation (5).

Several scholars among many have proposed varying conceptual frameworks in order to elucidate the complex nature of health system resilience. In the aftermath of the Ebola crisis, Kruk et al responded to the growing demands of multilateral organisations to illustrate five key characteristics of a resilient health system and a proposed resilience index corresponding to these five characteristics to measure resilience (6). Blanchet et al proposed a new model of resilience as an underlying management and governance capacity to absorb, adapt and transform itself in case of a shock (7). Gilson et al and Barasa et al introduced the idea of 'everyday resilience,' highlighting the capacities and resources that are required when delivering health services every day, which have to be built over a longer period to address chronic deficiencies in the health system (8), (9). Building on these ideas, Grimm et al have synthesized empirical studies from low- and middle- income countries to confirm Kruk et al's five characteristics of resilience and to identify five new characteristics that can serve as foundations to be prioritized in resource-constrained settings to unlock system-level resilience (10).

Critical in completing the resilience discourse especially in low- and middle- income countries is the role of the communities. A few empirical studies have revealed the vital role community engagement plays in building trust in the health system, which is in turn intricately linked with effective management of crises (11), (12), (13).

Cyclone Nargis and its legacy for Myanmar

A category three Cyclone Nargis struck Myanmar on the 2nd of May, 2008, affecting more than 50 townships and devastating mainly the Ayeyarwady Division and its capital, Yangon. It is recorded as Myanmar's worst natural disasters in recorded history (14). Within this area, the official death toll was estimated 138,000 (15), but the damage had severely affected at least 2.4 million lives in the region. Not only did the cyclone result in a widespread destruction of homes and major infrastructure, but close to 75% of the health facilities were damaged in the affected townships. Most of the destroyed facilities were that of primary health care, which exacerbated healthcare access to the rural poor and amplified economic inequalities (14), (16). The Burmese

For peer review only - http://bmjopen.bmj.com/site/about/guidelines.xhtml

government at the time missed the critical period following the cyclone to implement essential measures to save lives and provide relief efforts (17). The agreement to issue visas for foreign humanitarian aid workers and allow international assistance came three weeks after the storm, only after the UN Secretary-General Ban Ki-moon visited Yangon to plead with General Than Shwe, the head of the military state (18). To this day, the response to Cyclone Nargis reverberates lessons for the international community.

Objectives

The aim of this study is to understand how the characteristics of health system resilience have played out in Myanmar's response to Cyclone Nargis and to explore ways to improve resilience at the system level. Using a refined conceptual framework for health system resilience developed previously, this paper analyses Myanmar's assets and barriers to achieving health system resilience and highlights the lessons learnt from its devastating past with the hope that the lessons may be transferrable to other countries (10).

METHODS

Study setting

This is an explanatory qualitative study exploring the institutional capacity of resilience in Myanmar's health system. Myanmar was recently ranked second on the Global Climate Risk Index of countries most affected by extreme weather events from 1999 to 2018 (19), and 17th out of 191 countries on the Index for Risk Management (INFORM), which assesses the risk of humanitarian crisis and disasters that could overwhelm national capacity to respond (20). It is prone to various natural hazards such as earthquakes, floods, cyclones, droughts, and fires, which have intensified with the effects of climate change (21).

Myanmar's national health plan (2017-2021) highlights Universal Health Coverage (UHC) as its overarching goal and the Ministry of Health and Sports have initiated steps to improve compliance to the International Health Regulations (IHR) in the recent years (22), (23). Initiated by the Union Minister of Health and Sports, Myanmar was the third country in the Southeast Asia region to take part in the Joint External Evaluation (JEE) in 2017 to assess the country's capacity under the IHR to prevent, detect, and respond to public health threats (24). In order to follow-up on the recommendations from the JEE findings, Myanmar's Ministry of Health and Sports have developed a five year National Action Plan for Health Security (NAPHS) (25). These efforts demonstrate the country's political will to embark on a journey towards building the foundations of health system resilience.

Study design and participants

The study comprised of 12 in-depth interviews conducted between January and February 2020, with its participants purposively selected through a snowball sampling of relevant referrals via contacts introduced through the author PYG's existing network whilst working and living in Myanmar (26). This proved as an effective method to track participants that had been directly involved in the response efforts of Cyclone Nargis in 2008. Among the many stakeholder groups, only those who had represented or currently represents international organisations and non-governmental organisations have been included in the interviews. There was a balanced distribution of participants across UN, bilateral, and NGOs. Most of them were either Myanmar citizens or expatriates with extensive working experience based in Myanmar. As the interviews targeted a single stakeholder group, the 12 interviews were shown sufficient to reach data saturation (27).

For peer review only

For peer review only - http://bmjopen.bmj.com/site/about/guidelines.xhtml

Data collection

The interviews lasted between 1-1.5 hours respectively and were conducted in-person by the author PYG (PhD Candidate) in English. There were no prior exchanges between the participants and the data collector and the interviews were held in private office spaces of the participants. A semi-structured interview guide was developed to explore the roles of the participant, actions taken as an organisation, and perceptions of the strengths and weaknesses in the health system at the time of the crisis. The questions were framed around Kruk et al's five dimensions of resilience (5). See supplementary file for the full interview guide. All interviews have been audio-recorded following informed consent and thereafter transcribed verbatim. Interviewees were approached a second time via video conference in order to verify the accuracy of the transcripts.

Data analysis

Analysis proceeded using a data-driven thematic analysis, closely following the steps of the framework method (28). Verbatim transcriptions and field notes were reviewed for familiarization and verified for accuracy. Following the development of an initial codebook inspired by the ten themes previously developed by the author's team, all transcripts were coded line-by-line using MAXQDA 2018. A hybrid of deductive and inductive approaches provided for a broader structure of the categories and flexibility of codes from the open coding process. Grimm et al's conceptual framework was used to develop the analytical framework (10). The framework was then applied systematically to chart the relevant summary of the transcript with direct quotations structured around 10 categories and 21 codes, resulting in a complete framework matrix. This process enabled comparisons and contrasts of key emergent themes between the participants, which later generated key results describing the foundational assets, barriers and opportunities for achieving resilience in Myanmar. A few of the interviewees provided feedback on the final analysis through a subsequent virtual meeting arranged by the author.

Ethical considerations

The proposal for this study was approved in Myanmar by the Institutional Review Board of the University of Public Health and the Ministry of Health and Sports of the Republic of the Union of Myanmar (2020/Research/4). It has also received clearance in Switzerland by the Ethikkommission Nord-west und Zentralschweiz (Req-2019-00348).

Patient and public involvement

The research was done without public involvement.

RESULTS

The results section presents key findings elucidating the characteristics of resilience that have been salient in Myanmar's response to Cyclone Nargis. The results are categorised into assets, barriers, and future opportunities for achieving health system resilience.

Foundational assets that can unlock Myanmar's health system resilience

Strong social capital comprised of informal channels and trusted networks

Most participants pointed to the extensive role that the informal sector plays in filling the gaps of the health system. A case in point, the ambulance system in Myanmar is charity-based and

For peer review only - http://bmjopen.bmj.com/site/about/guidelines.xhtml

For peer review only

subjected to resource availability from various donors. The township hospitals themselves have to seek out appropriate charity groups to maintain the referral system comprised of ambulances and drivers. Likewise, when Cyclone Nargis hit, there were various community-based organisations as first responders of the crisis, offering food, shelter and basic commodities to the affected villages. Strong social capital in Myanmar were exhibited mainly through informal channels, whether it be via monasteries and community-based charity groups. They were able to operate without legal mandates and special memorandum of understanding, precisely because they have obtained people's trust and leveraged the familiarity of their own network. One NGO worker described:

> *"There was an existing social community that was ready to donate to religious events and they were actively involved and participated in the relief efforts after Nargis hit. Each village has a social committee and the religious leaders in the monastry have their own shelters for the affected people who sought refuge. The communities themselves know their needs and gaps the best, which can build resilience." (I09)*

In a context where access to the affected regions were limited for the first three weeks after the cyclone, community engagement proved vital in offering timely support. It was apparent that participants who visited the affected areas experienced severe logistical challenges. Some of the areas were only accessible by boat or in off-road conditions where a robust 4x4 vehicle was required. In this endemically challenging environment, mobile clinics were established to reach the communities in remote areas. All the more in these contexts, community-based resilience was not optional but imperative.

> *"People had to travel long distances to access health care, which severely limited preventative care and people accessing the health system…One thing that consistently stood out to me was the incredible self-sufficiency of the communities themselves, their resilience, and their ability to get back on their feet with the limited resources that they had." (I11)*

Referring back to the example of charity-based ambulances, a few participants stated their concerns that this heavy reliance on informal channels can in turn be a barrier towards building basic systematic preparedness, as often these groups can run without standardized procedures and beget further inequalities in service delivery.

> *A basic preparedness however is not equipped because of this reliance on informal channels. Not all township hospitals have ambulances. Apparently, there is no standard. An ambulance and a driver are not part of a township health department set up. (I06)*

As evidenced through Cyclone Nargis, social capital albeit informal in nature served as an asset for Myanmar in both crisis and calm.

Cultural and religious commitment as a propeller for motivation

Underpinning the strong social capital lay the commitment of community-based groups as the backbone of relief efforts. Many attested that Myanmar's history of prolonged isolation and under-development led to a stronger demonstration of resilience at the community level especially in a situation of emergency. In Myanmar, volunteerism is a common method of civic participation (29). The motivation for this commitment appeared to have stemmed from the people's religious and cultural beliefs to give to charity and donate to the less fortunate, which also manifested in the case of Nargis. A UN staff member shared:

> *"People in a community supporting other people in need is a powerful response. Most believe that it's because they were closed off for so long, and it was just helping themselves, and the nature of the general beliefs and teachings of the country. People will go to churches, pagodas, temples, monastries or other public buildings to shelter, and the community there will provide food for a couple of days generally." (I07)*

For peer review only - http://bmjopen.bmj.com/site/about/guidelines.xhtml

Traditional social welfare support groups revolving often through informal channels played a vital role at the village level and many self-help groups sprung up spontaneously to join the momentum (30). The monasteries even had an informal system to store food collected through donations and shared them with the communities in need. They were the sole sources of aid in the immediate aftermath of Cyclone Nargis until support arrived from the government and UN agencies, which proved as a clear asset. Several interviewers echoed that these social determinants of religion and culture provided a supporting environment for subsequent relief projects to flourish.

> *"The Buddhist culture compels people to donate to those in need and the monks. The first responses have always come from the people/monasteries, later from the government and finally from the UN agencies. It's because it's part of the culture, all these donations." (I01)*

> *"There are social determinants at play in the response to Nargis. At the time of the disaster, 90% of the people were Buddhist. Those from non-affected townships even came. Came to donate. This is the culture of Buddhism. Supporting environment was religion." (I05)*

Community resilience in Myanmar seems to have been built on a stronger foundation because it stems from religion and culture as a motivating force. The powerful source of solidarity within some of the affected communities weathered through generations of political and ethnic divisions.

Barriers to Myanmar's journey towards resilience

Historical legacy affecting transparency, openness and decentralisation

When reflecting upon the aftermath of the cyclone, all participants called out the lack of transparency and openness in how the government had dealt with the crisis at hand. The government at the time did not convey the exact situation analysis to the public, withheld key information about the cyclone, and controlled data such as the degree of damage and casualties incurred. Such lack of transparency not only undermined public trust towards the government but also limited international response teams from accessing Myanmar, which resulted in an overall delay in collective response efforts.

> *"The government at that time was so quiet and the civil society has not seen the first response from the government. INGOs were not able to access the area until three weeks after the cyclone hit. Perhaps, this is the reason why the government also tried to hide the information as much as possible, for instance, the number of casualties, number of shelters required, etc. We learnt the lack of government information is tied to the lack of accountability." (I10)*

Though the reasons may be complex, most respondents pointed to Myanmar's long history of military rule that has operated based on a rigid hierarchy and centralized decision-making system, a context that may not have been conducive for disaster management. There was no proper disaster management mechanism in place; rather, coordination was led through the health cluster. As it had been a highly centralized system, however, the senior general still had the final say and was initially not open to collaborating with the outside world.

> *"Everything had to be reported and decided by the senior general (Than Swe)..Whatever he said, everybody has to do it." (I04)*

To this day, the Memorandum of Understanding (MoU) is signed at the national level, not with the state. The requests may be made locally, but the final say comes centrally. One participant stated that this hierarchical structure produces dissonant voices at different levels, complicating the decision-making process and posing a risk of not meeting the actual needs on the ground. Another participant echoed this sentiment:

For peer review only - http://bmjopen.bmj.com/site/about/guidelines.xhtml

> *"Myanmar is too centralized, which poses a key challenge towards building a resilient health system. Evidence-based planning at the local level is not possible. For example, prevention of the polio outbreak that occurred in Hpa-bun would have been possible if local collaboration with the ethnic organisation could be done at the state level, where states have the decision making power and have fiscal decentralisation." (I06)*

An added layer of complication is the presence of ethnic armed groups in self-administered zones that are run independently of the government as a result of its ongoing strife towards independence (31). These special subnational administrations run by multiple ethnic groups not only have their own health system parallel to that of the Myanmar government but selectively collaborates with local NGOs and community-based groups where there are no conflicts of interest. One participant shared that the recent polio outbreak in one of these self-administered regions was particularly difficult to contain due to lack of information shared and restricted access to the area.

> *"Often ethnic health organisations work in these areas to provide services, and some are in fact coordinating well with the ministry. But, access can be difficult, as in the case of the polio outbreak, and the ethnic health organisation wouldn't let the ministry in nor UN agencies. They only allow one NGO they trust to have access. " If you're looking at some kind of outbreak, there is a needed preparedness element by the ministry, but they also need to consider this other factor that is not something that other countries should necessarily worry about." (I06)*

Often UN agencies and NGOs offer to bridge these gaps in services, yet these two parallel systems pose great challenges for international actors to maneuver through the bureaucracies in these conflict zones. It is an ongoing challenge that Myanmar needs to overcome in order to reach an inclusive state of resilience at the national level.

Lack of investments in long-term variables of resilience

Cyclone Nargis revealed the fragility of its health system, especially the areas that had required long-term investments. One participant that had worked with the UN agency during this time depicted how dire the situation had been.

> *"The basic fabrics of the health system were not existing. There was not a place to even fit supplies, medicines and computers. Many RHCs were not functioning well, many times lacking midwives and essential medicine. The nurse to patient ratio was too low and building the nursing infrastructure was critical." (I04)*

According to these first-hand accounts, Myanmar still had a very basic infrastructure, township hospitals covering up to 200,000 people, and a rural health centre serving around 20,000-25,000 people (14). A typical rural health centre had a midwife but this was not necessarily the case in remote and hard-to-reach villages. Health worker shortages came up in all the interviews as a major obstacle for Myanmar's achieving its Universal Health Coverage goal, let alone its journey towards resilience. One UN staff indicated that on average only 54% of the sanctioned health worker posts are filled in Myanmar and building a strong human resource cadre that is evenly distributed across the health system requires a conscious long-term commitment and investment from the government.

> *"Though development cooperation can support buildings, supplies and equipments, robust human resources require a long-term commitment and investment that should come from the government..I mean, the largest issue of Myanmar is that you have only 54% of sanctioned posts filled. Even the 54%, if you see differences in regions." (I06)*

All participants stressed the importance of investing in long-term infrastructure, including its health workforce across the country, particularly focusing on extending its health system coverage to remote and vulnerable areas in order to build resilience of the health system as a whole. However, the stark inequalities in remote areas and the frequent staff turnover at all

For peer review only - http://bmjopen.bmj.com/site/about/guidelines.xhtml

levels of the government were reflections of poor working conditions and weak human resource policies. Participants currently working for a major UN agency shared that funding additional trainings may not be the solution to this health workforce challenge.

> *"Because of the weakness in the human resource policy, and the working nature of the government job, many government staff are resigning, quitting their jobs. We are losing qualified candidates. Human resource is a major challenge...I know many trainings were supported by organisations and donors, but the problem is the retention of human resource. After the trainings, they move or resign. I think training should not be the key weakness." (I02)*

Some specifically referred to the importance of foresight for these long-term commitments. There seems to be a significant gap between plans on paper and implementation as mentioned by one civil society representative:

> *"The challenge in the state/region is human resource shortages, especially medical doctors. The analysis of the ministry of health national health plan reveals that across the country there is a health service gap due to the lack of human resources. There are not enough medical doctors trained through the medical universities. The government already has a plan, but there is a big implementation gap." (I10)*

Myanmar's future for resilience

Harnessing social capital to unleash the power of collective civic duty through civil society groups

As echoed by multiple participants, Myanmar appears to have the ingredients for resilience within its own respective communities. Not only did existing community-based groups act as first responders of Cyclone Nargis albeit decades of dictatorship, but even the coordination took place naturally and provided mutual support. It was simply the people exhibiting civic duty and playing their due role in the society. One representative of a local civil society group shared that health system resilience may be reached when civil society groups build community resilience and start working together with a health system that is also investing in building its strengths.

> *"At the time of the cyclone, the local civil society groups in each area organised and coordinated with each other by themselves. We haven't seen any government, like red cross, or police. We haven't seen any solider. During the Cyclone Nargis, we have seen the communities, the people, how did they respond. They came out to the street and they supported each other. They tried to remove all the trees and blocks. We learnt from them. We have strong resilient communities. Communities know best what the biggest challenge and needs are in those areas. If we work together, community resilience strengthening and health system strengthening, we could be working towards resilience." (I10)*

The informal channels of self-formed community-based groups can be a strong basis for the institutionalization of civil society groups that have a rather complicated relationship with the government. Since Myanmar has opened up in 2011 under President Thein Sein, its reform process has gradually enabled a platform for civil society to establish civil liberties through its '2008 Constitution,' with the article 354 stating the right to freedom of expression, assembly and association (32). Furthermore, the Parliament has enacted the Association Registration Law, which defines and clarifies local and international non-governmental organizations and stipulates requirements to register with the government. These reforms have gradually shifted the landscape of civil society engagements at all levels of the country.

Even at the national level, the government has been more receptive to the inputs of the civil society groups, including the Ministry of Health and Sports with many of its community-driven development projects. There are certainly growing opportunities for civil society groups to participate in the various development sector working groups (33). One participant

For peer review only

For peer review only - http://bmjopen.bmj.com/site/about/guidelines.xhtml

reflects on the evolution of civil society engagement since Cyclone Nargis and projects that no crisis can be handled without the involvement of the country's civil society groups.

> *"The health system at that time was already weak and the ministry of health didn't have a strong coordination with the civil society or community groups. The government organised many meetings but had no idea how to work with the local civil society. Only in the last four years did this relationship change. The minister of health stressed the importance of working with CSOs across the country. If an outbreak were to happen, the ministry of health cannot handle the situation without civil societies." (I10)*

Connecting its overarching health goals with long-term investments to improve resilience

As Myanmar is situated in a disaster-prone area, it has naturally developed a local expertise over time through handling its yearly floods. One participant stated that there is an emergency fund stipulated by law to set aside a disaster management fund that can be used to support sub-national levels in case of a potential crisis. How the legal frameworks and plans align with its budgeting and allocation, however, is a different issue.

A recurring theme that transpired when discussing the government's response to the cyclone and other subsequent crises were the emergence of discrepancies between planning, budgeting and implementation. As there has been no clear alignment between its long-term health policy and crisis management, the targeted investment towards building system-level resilience has also lagged behind.

> *"There is very remote connection between planning and budgeting. It happens both at the national and local levels. Preparedness is there on paper and so far there were no big loss in human lives." (I06)*

One participant called this the ability to "expand and contract," which also corresponds to the concept of adaptiveness in Kruk et al's framework. It is exhibited by the ability to reallocate its resources to where it is most needed on time and to delegate the authority to the appropriate decentralized administration when necessary (6). It is a vital characteristic of resilience, which is a byproduct of long-term investments and painstaking reforms (34).

> *"This kind of planning, the constant expansion and contraction that you would need in an environment like Myanmar that's so natural disaster heavy, is difficult." (I07)*

Many participants agreed that Myanmar ought to invest towards building long-term infrastructure especially in remote areas and prioritize in its human resource preparedness in order to build resilience in the system. This may involve employing creative ways to increase staff retention through the engagement of the private and non-profit sectors. More importantly, however, these plans ought to connect back to its overarching national goal for Universal Health Coverage and its momentum to strengthen its commitment towards International Health Regulations.

> *"In order to improve health system resilience, it is necessary to improve infrastructure needed at the village level… A challenge in building the long-term capacity of health workers, however, is the frequent turnover of staff. Mostly staff assigned for the rural health centres are based there for 1-2 years and move to a better place. Turnover was quite quick." (I09)*

DISCUSSION

This study explored the degree to which key characteristics of health system resilience manifested in Myanmar's response to Cyclone Nargis and their respective historical and socio-political determinants. As health systems are complex and evolving in nature, it is important to predicate this study on the view that resilience is a capacity that can be nurtured with time and commitment (35). Moreover, facets of resilience should not be seen as

For peer review only - http://bmjopen.bmj.com/site/about/guidelines.xhtml

disconnected inputs, but rather a holistic result of targeted investments and a series of structural reforms (10), (34).

In the case of Myanmar, it had been the ‘everyday resilience’ of its frontline workers and communities that had been built through its day-to-day chronic struggles in the context of its weak governance and political and economic vulnerabilities, that in turn prepared them for the unexpected shock (8). As depicted in Figure 1, key themes that emerged from the analysis have been organised into assets, barriers and future opportunities in describing Myanmar’s journey towards resilience.

Figure 1. Assets, barriers and opportunities for Myanmar’s journey towards health system resilience

First, social capital and motivation have surfaced as key foundational assets in Myanmar that are indicative of its community resilience. The power of collective civic duty filled the service gaps that the government had failed to provide in the immediate aftermath of the cyclone. The social capital present in Myanmar during Cyclone Nargis were comprised of informal channels and trusted networks in the communities, its motivation stemming from a deeper faith rooted in religion and culture. A recent qualitative synthesis highlighted social capital as the interconnecting mechanism common to both health system strengthening and resilient responses to crises, a powerful tool that can unlock resilience of the health system (10). This study extends the notion a step further by demonstrating that the power of social capital can go beyond its health system capacity. Social capital may in fact play a more important role in providing relief from the cyclone, just as it had been reported as being more vital in mitigating the spread of the Ebola virus than any donor-driven short-term interventions (13). The pivotal role social capital plays in building community resilience have been well documented by other studies in the context of both natural and man-made disasters (36), (37), (38), (39). When communities themselves start carrying out critical health system functions, this collective momentum for community resilience prompts what Barker et al calls a ‘fortuitous cycle’ of increased trust, improved communication and continued meaningful community engagement, all of which are crucial preconditions for enabling health system resilience (11).

Second, Myanmar’s historical legacy of prolonged military rule and a lack of long-term investments posed as major barriers to achieving resilience. The lack of transparency and openness stood out as the biggest obstacle towards crisis management, which undermined public trust towards the government and hampered international response teams from accessing Myanmar in the initial stages. Myanmar is a prime example of a post-colonial state which development experts describes as a ‘conflict trap’ (40). The legacy of the colonial experience, ethnic insurgencies, and a successive military rule, shifted all of its government’s priorities to regaining its national sovereignty and ensuring security through increased military spending (41). Consequently, little had been left for health and social welfare, with only approximately 2% of its GDP spent on health care shortly before the cyclone hit (30). It is no surprise that Myanmar’s health system was fragile and not ready to handle a disaster of such magnitude.

Governance practices influence organizational resilience and decentralized governments are certainly at an advantage to exhibit the necessary flexibility and timely responses required in crisis management (2). Having a decentralized governance structure itself, however, does not guarantee a successful response. China had no national disaster management agency nor related policies during the Sichuan earthquake, but effectively controlled the emergency with its President’s charismatic leadership and vertical administrative structure (42). Myanmar’s

For peer review only - http://bmjopen.bmj.com/site/about/guidelines.xhtml

centralized and hierarchical system could have been used for Myanmar's advantage, but an adequate disaster management had not been the priority of the government.

A common pattern across failed attempts in responding to a crisis was to prioritize on the 'fast variables,' delivering services in the form of temporary shelters and supplies without considering its longer-term infrastructure building, as it had been in the case of Liberia during its Ebola crisis (43). Building resilience, however, is much more than preparedness, as Kruk et al states, that it requires investing in systems and its corresponding 'slow variables' that can function in both crisis and calm (6). A health system that has a clear foresight for its people will strengthen its health system infrastructure and train its human resource cadre to build the foundations of its health system (10). In the context of Myanmar, it would mean to apply a systems thinking approach in examining its past shortcomings and redesigning its institutions and organisational structures to serve its evolving purpose (44).

Third, Myanmar may channel its assets into opportunities by using its growing civil society platforms and leveraging its long-term national health goals. Cyclone Nargis revealed that Myanmar has the key ingredients for resilience within its own communities through its people exhibiting their civic duty. Civil society has had a long history in Myanmar dating back to its informal Buddhist associations in the traditional kingdoms and various other religious groups organizing social and welfare programmes during its colonial period and beyond (45). Since Cyclone Nargis, the space for civil societies further expanded through its legal reforms but at the end of the day only a few have been able to obtain official registration and secure a sustainable funding mechanism (45). According to the Civil Society Survey conducted in 2019, despite its marked progress, a wide range of obstacles still remain and hinder civil societies from reaching their goals (32). Myanmar has set ambitious national goals to reach Universal Health Coverage (UHC) and to improve compliance to the International Health Regulations (IHR) in order to ensure healthcare and health security for all (22), (25). As evident in its neighbouring countries, however, civil society engagement and community feedback loops for policy and decision-making processes have shown as prerequisites to accelerate progress towards achieving these compendium of goals (46). Trust and accountability at all levels in the system are what builds the 'capacity of legitimacy,' a crucial component of resilient health systems, that can only be strengthened through an inclusive consultation process of engaging communities and civil society groups (7). Myanmar has to realize that strategically leveraging these opportunities may be the fastest route to building resilience.

Study Strengths and Limitations

This study is empirical in nature, building on a health system resilience conceptual framework developed from a previous systematic review. The framework has been tested for the first time for its applicability in a low- income country. The topic of health system resilience has a potential to make a timely contribution to the health systems and policy research community, with lessons generated that can be transferrable to other contexts. Due to Covid-19 pandemic's travel restrictions imposed during the data collection period, the study only represents the view of international organisations and non-governmental organisations, restricting the scope of the study to the perceptions of a single stakeholder group. Further studies should explore capturing a wider range of stakeholders, the perceptions of communities, frontline workers and government staff that may be able to offer a more holistic view of the phenomenon.

CONCLUSION

For peer review only

This study revealed that resilience in the health system can be built through targeted investments towards building the foundations of resilience to better prepare for future shocks. In the case of Myanmar, social capital and motivation, which surfaced as its foundational assets, may be channelled into opportunities that can help achieve its long-term health goals, accelerating its journey towards resilience in the health system.

ACKNOWLEDGMENTS

The authors would like to extend appreciations to all the interviewees who carved out time to part take in this study. Special thanks to Dr. Nilar Tin for her valuable input on the initial study concept and for suggesting relevant interviewees.

CONTRIBUTORS

PYG and KW conceptualized the study; PYG collected and analysed the data with substantive input from SM and KW. PYG developed the first draft of the manuscript and all authors contributed to the subsequent and final drafts.

FUNDING

This article is part of the PhD project that has received funding from the European Union's Horizon 2020 research and innovation programme under the Marie Skłodowska-Curie grant agreement No. 801076, through the SSPH+ *Global PhD Fellowship Programme in Public Health Sciences (GlobalP3HS) of the Swiss School of Public Health*. The corresponding author is also a recipient of the Swiss Government Excellence Scholarship (ESKAS), provided by the Swiss Federal Commission for Scholarships for Foreign Students (FCS) with the award number 2018.0217. The funding bodies did not play a role in the design, analysis, and writing of the manuscript.

COMPETING INTERESTS

None declared

PATIENT CONSENT FOR PUBLICATION

Not required

ETHICS APPROVAL

The proposal for this study was approved in Myanmar by the Institutional Review Board of the University of Public Health and the Ministry of Health and Sports of the Republic of the Union of Myanmar (2020/Research/4). It has also received clearance in Switzerland by the Ethikkommission Nord-west und Zentralschweiz (Req-2019-00348).

DATA AVAILABILITY STATEMENT

Data are available on reasonable request and approval by the authors and involved project partners.

REFERENCES

1. Global HS. Vancouver statement for the fourth global symposium on health systems research. 2016.
2. Barasa E, Mbau R, Gilson L. What Is Resilience and How Can It Be Nurtured? A Systematic Review of Empirical Literature on Organizational Resilience. International Journal of Health Policy and Management. 2018;7(6):491-503.

For peer review only

For peer review only - http://bmjopen.bmj.com/site/about/guidelines.xhtml

3. Fridell M, Edwin S, Von Schreeb J, Saulnier DDJIJoHP, Management. Health system resilience: what are we talking about? A scoping review mapping characteristics and keywords. International Journal of Health Policy and Management. 2020;9(1):6.
4. Biddle L, Wahedi K, Bozorgmehr KJHP, Planning. Health system resilience: a literature review of empirical research. 2020.
5. Kruk ME, Myers M, Varpilah ST, Dahn BT. What is a resilient health system? Lessons from Ebola. The Lancet. 2015;385(9980):1910-2.
6. Kruk ME, Ling EJ, Bitton A, Cammett M, Cavanaugh K, Chopra M, et al. Building resilient health systems: a proposal for a resilience index. BMJ. 2017:j2323.
7. Blanchet K, Nam SL, Ramalingam B, Pozo-Martin F. Governance and Capacity to Manage Resilience of Health Systems: Towards a New Conceptual Framework. Int J Health Policy Manag. 2017;6(8):431-5.
8. Gilson L, Barasa E, Nxumalo N, Cleary S, Goudge J, Molyneux S, et al. Everyday resilience in district health systems: Emerging insights from the front lines in Kenya and South Africa. BMJ Global Health. 2017;2.
9. Barasa EW, Cloete K, Gilson L. From bouncing back, to nurturing emergence: reframing the concept of resilience in health systems strengthening. Health Policy Plan. 2017;32:91-4.
10. Grimm PY, Oliver S, Merten S, Han WW, Wyss K. Enhancing the Understanding of Resilience in Health Systems of Low- and Middle-income Countries: A Qualitative Evidence Synthesis International Journal of Health Policy and Management. 2021:-.
11. Barker KM, Ling EJ, Fallah M, VanDeBogert B, Kodl Y, Macauley RJ, et al. Community engagement for health system resilience: evidence from Liberia's Ebola epidemic. 2020;35(4):416-23.
12. Bhandari S, Alonge OJHrp, systems. Measuring the resilience of health systems in low-and middle-income countries: a focus on community resilience. 2020;18(1):1-19.
13. Alonge O, Sonkarlay S, Gwaikolo W, Fahim C, Cooper J, Peters DJGha. Understanding the role of community resilience in addressing the Ebola virus disease epidemic in Liberia: a qualitative study (community resilience in Liberia). 2019;12(1):1662682.
14. Group TC. Post-Nargis Joint Assessment. 2008.
15. EM-DAT: The Emergency Events Database [Internet]. Universite catholique de Louvain (UCL). Available from: www.emdat.be.
16. Warr P, Aung LLJWD. Poverty and inequality impact of a natural disaster: Myanmar's 2008 cyclone Nargis. 2019;122:446-61.
17. Stover E, Vinck PJJ. Cyclone Nargis and the politics of relief and reconstruction aid in Burma (Myanmar). 2008;300(6):729-31.
18. Secretary-General's press conference at Hotel Sedona in Yangon [press release]. 23 May 2008.
19. David Eckstein VK, Laura Schäfer, Maik Winges. Global climate risk index 2020 Germanwatch; 2019.
20. INFORM Global Risk Index 2020 [Internet]. European Commission 2019. Available from: https://drmkc.jrc.ec.europa.eu/inform-index/.
21. UNOCHA. HCT Myanmar- Inter-agency emergency response preparedness (ERP) plan 2017.
22. Ministry of H, Sports tRotUoM. Myanmar National Health Plan 2017-2021. 2016.
23. Pyone T, Aung TT, Endericks T, Myint NW, Inamdar L, Collins S, et al. Health system governance in strengthening International Health Regulations (IHR) compliance in Myanmar. 2020;5(11):e003566.
24. Joint external evaluation of IHR core capacities of The Republic of the Union of Myanmar: executive summary. . World Health Organization; 2017
25. National Action Plan for Health Security (NAPHS) 2018-2022. Ministry of Health and Sports, the Republic of the Union of Myanmar 2018.
26. Naderifar M, Goli H, Ghaljaie FJSiDoME. Snowball sampling: A purposeful method of sampling in qualitative research. 2017;14(3):1-6.

For peer review only - http://bmjopen.bmj.com/site/about/guidelines.xhtml

27. Guest G, Bunce A, Johnson L. How Many Interviews Are Enough?: An Experiment with Data Saturation and Variability. Field Methods. 2006;18(1):59-82.
28. Gale NK, Heath G, Cameron E, Rashid S, Redwood S. Using the framework method for the analysis of qualitative data in multi-disciplinary health research. BMC Medical Research Methodology. 2013;13(1).
29. Dove CJAJoS-EAS. Giving Trends in Myanmar: More Than Merit Making. 2017;10(2):205-22.
30. The Republic of the Union of Myanmar Health System Review: World Health Organization; 2015 2015.
31. Jolliffe KJTAF. Ethnic armed conflict and territorial administration in Myanmar. 2015:89.
32. Buschmann A, Soe AJAaS. Authoritarian Impediments to Civil Society in Contemporary Myanmar: Findings from the Myanmar Civil Society Survey 2019. 2020.
33. Civil society briefs: Myanmar. Asian Development Bank 2015.
34. Kutzin J, Sparkes SPJBotWHO. Health systems strengthening, universal health coverage, health security and resilience. 2016;94(1):2.
35. Topp SM. Power and politics: the case for linking resilience to health system governance. BMJ Specialist Journals; 2020.
36. Bianca RJTRoAS. Influence of social capital on community resilience in the case of emergency situations in Romania. 2018;14(54):73-89.
37. Aldrich DP, Meyer MA. Social Capital and Community Resilience. American Behavioral Scientist. 2015;59:254-69.
38. Pfefferbaum B, Van Horn RL, Pfefferbaum RLJCSWJ. A conceptual framework to enhance community resilience using social capital. 2017;45(2):102-10.
39. Aldrich DP. The importance of social capital in building community resilience. Rethinking resilience, adaptation and transformation in a time of change: Springer; 2017. p. 357-64.
40. Smith M. State of strife: The dynamics of ethnic conflict in Burma: Institute of Southeast Asian Studies; 2007.
41. Than TMM. Myanmar: preoccupation with regime survival, national unity, and stability. Standford: Stanford University Press; 1998. 390-416 p.
42. Lin L, Ashkenazi I, Dorn BC, Savoia E. The public health system response to the 2008 Sichuan province earthquake: a literature review and interviews. Disasters. 2014;38(4):753-73.
43. Ling EJ, Larson E, Macauley RJ, Kodl Y, VanDeBogert B, Baawo S, et al. Beyond the crisis: did the Ebola epidemic improve resilience of Liberia's health system? Health Policy Plan. 2017;32(suppl_3):iii40-iii7.
44. Arnold RD, Wade JPJPCS. A definition of systems thinking: A systems approach. 2015;44(2015):669-78.
45. Kramer T. Civil society gaining ground: Opportunities for change and development in Burma: Transnational Institute Amsterdam; 2011.
46. Yeoh E-K, Johnston C, Chau PYK, Kiang N, Tin P, Tang JJHS, et al. Governance functions to accelerate progress toward universal health coverage (UHC) in the Asia-Pacific Region. 2019;5(1):48-58.

For peer review only

For peer review only - http://bmjopen.bmj.com/site/about/guidelines.xhtml

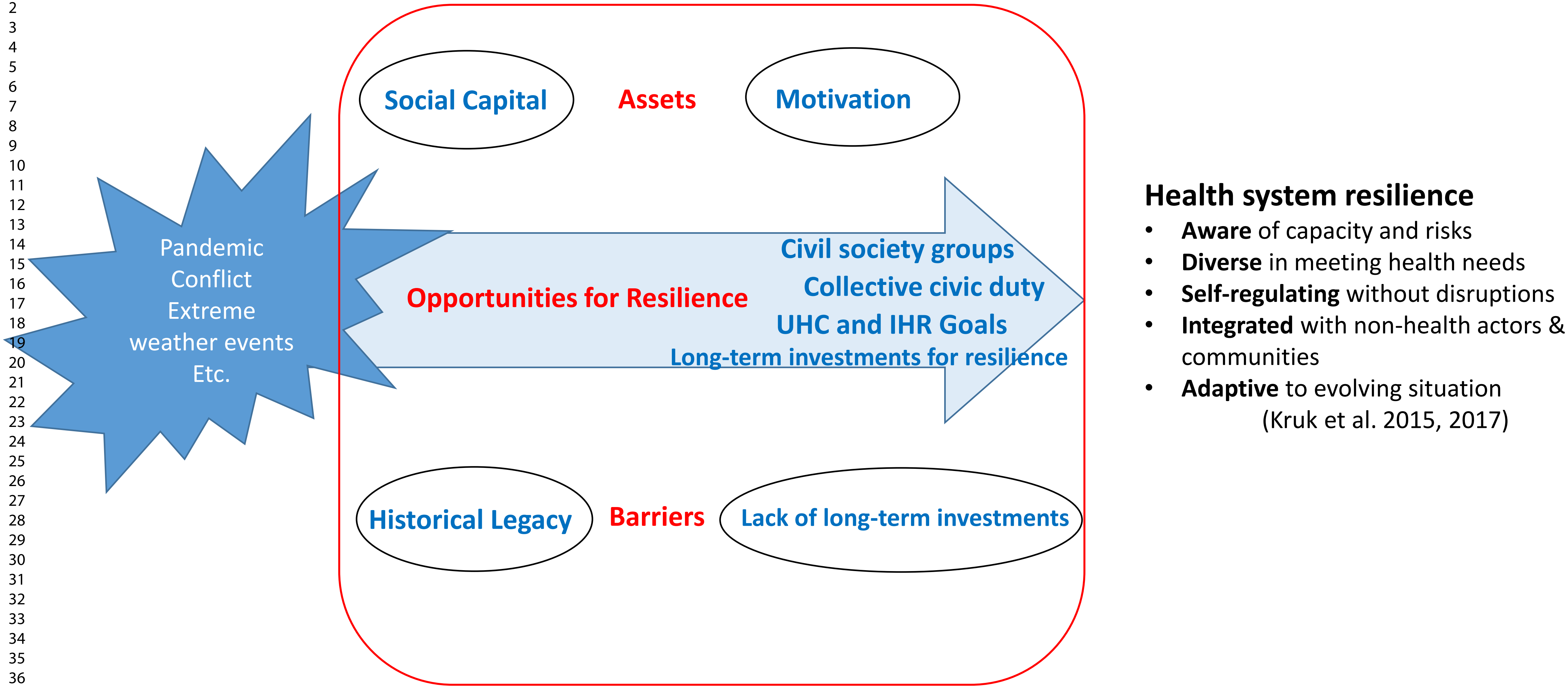

Figure 1. Assets, barriers and opportunities for Myanmar's journey towards health system resilience

SUPPLEMENTARY FILE. INTERVIEW GUIDE

Study Title: Health System Resilience: key for unlocking the complex health system strengthening agenda

Investigator: ________________________ **Phone number:**___________________________

Organisation/Department: ______________________________ **Date: |__|__|__|__|__|__|**

Position/Title: ____

Stakeholder Group:

□ Central Government

□ Local Government (Township, local health committees, member of parliament, etc)

□ Health workers

□ Int'l Agencies (UN, NGOs, Bilateral, etc)

Number of participants: |__|__|

DRAFT SEMI-STRUCTURED IN-DEPTH INTERVIEW QUESTIONS

Introduction: purpose of research, use of audio tape recorder, confidentiality

I. PARTICIPANT INTRODUCTION

1. What is your role when the country is hit with an external shock, whether it be a natural disaster, unforeseen pandemic, etc.

Prompt: examples of disaster management, pandemic, natural disasters

II. CASE 1: CYCLONE NARGIS (2008)

2. (General) Can you please describe in detail the account of Cyclone Nargis?

Probe: What was your title/position at the time?

What was the response protocol (written or perceived norm)?

What actions (out of the norm) did you take?

How did you respond as an individual?

3. (General) Can you please describe in detail the response actions taken as an organisation that you were a part of at the time?

Probe: Decision-making protocol? Evidence?

For peer review only

4. (General) Can you comment on one strength and weakness that the health system exhibited during Cyclone Nargis?

Probe: Which key element was present or missing during the incident?

5. (Dimension 1_Aware) At the time of Cyclone Nargis, how was the health system monitoring and tracking its health risks?

6. (Dimension 1_Aware) Once the disaster hit, who was included in the communication channel?

7. (Dimension 1_Aware) Who made the decisions regarding resource allocation and coordination efforts?

8. (Dimension 2_Diverse) At the time of Cyclone Nargis, were a range of health services still offered in health facilities (business as usual) or was the quality/quantity of services compromised? Why?

9. (Dimension 3_Self-regulating) Can you recall the extent of non-state organisations intervening to provide support at the time of crisis? (e.g. NGOs, Int'l Orgnisations, private actors, etc)

10. (Dimension 3_Self-regulating) At national level, were there reserve capacity (budget, HR, other resources) to summon quickly?

11. (Dimension 4_Integrated) Can you comment on the level of coordination between government, global and private actors at the time of the crisis?

12. (Dimension 4_Integrated) Who was the main actor responsible for coordination?

13. (Dimension 5_Adaptive) Was there at the time flexibility when it came to spending budget to respond to the emergency?

14. (Dimension 5_Adaptive) Can you comment on how much decision-making was possible at the decentralised/local level?

15. What was the main lessons learnt after undergoing Cyclone Nargis?

III. CONCEPT OF HEALTH SYSTEM RESILIENCE (HSR) AND GENERAL QUESTIONS

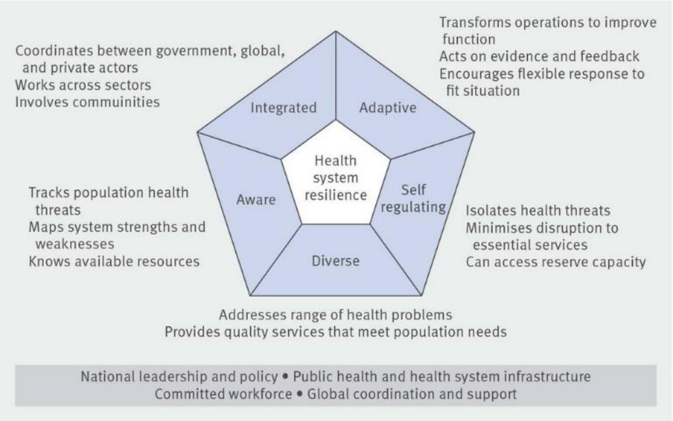

Figure 1. Resilient Health System Framework

16. Reviewing the framework above, how would you describe Myanmar's health system resilience generally?

Probe: Can you give concrete examples that indicate Myanmar's HSR?

17. What is Myanmar's strength/weakness when it comes to resilience?

18. What do you think is the most important element of resilience?

IV. PREPAREDNESS AND FUTURE SHOCKS

19. What may be the biggest threat/risk for Myanmar?

20. At this point in time, how would you describe Myanmar's preparedness for future shocks?

21. What national policies are in place to enable the Ministry of Health to be resilient to future shocks?

22. Can you comment on the efforts taken by the Ministry of Health and Sports to improve preparedness? (HR allocation, crisis management team, emergency plan, trainings undertaken, protocols, etc)

23. What would you advise to improve health system resilience in Myanmar?

Thank you for your time and heartfelt responses to the interview questions.

For peer review only

Consolidated criteria for reporting qualitative studies (COREQ): 32-item checklist

Developed from:
Tong A, Sainsbury P, Craig J. Consolidated criteria for reporting qualitative research (COREQ): a 32-item checklist for interviews and focus groups. *International Journal for Quality in Health Care*. 2007. Volume 19, Number 6: pp. 349 – 357

No. Item	Guide questions/description	Reported on Page #
**Domain 1: Research team and reflexivity**		
Personal Characteristics		
1. Inter viewer/facilitator	Which author/s conducted the inter view or focus group?	pg.4
2. Credentials	What were the researcher's credentials? E.g. PhD, MD	pg.4
3. Occupation	What was their occupation at the time of the study?	pg.4
4. Gender	Was the researcher male or female?	N/A
5. Experience and training	What experience or training did the researcher have?	pg.4
Relationship with participants		
6. Relationship established	Was a relationship established prior to study commencement?	pg.4
7. Participant knowledge of the interviewer	What did the participants know about the researcher? e.g. personal goals, reasons for doing the research	pg.4
8. Interviewer characteristics	What characteristics were reported about the inter viewer/facilitator? e.g. Bias, assumptions, reasons and interests in the research topic	pg.4
**Domain 2: study design**		
Theoretical framework		
9. Methodological orientation and Theory	What methodological orientation was stated to underpin the study? e.g. grounded theory, discourse analysis, ethnography, phenomenology, content analysis	pg.4
Participant selection		
10. Sampling	How were participants selected? e.g. purposive, convenience, consecutive, snowball	pg.3
11. Method of approach	How were participants approached? e.g. face-to-face, telephone, mail, email	pg.4
12. Sample size	How many participants were in the study?	pg.3
13. Non-participation	How many people refused to participate or dropped out? Reasons?	N/A

For peer review only

Setting		
14. Setting of data collection	Where was the data collected? e.g. home, clinic, workplace	pg.4
15. Presence of non-participants	Was anyone else present besides the participants and researchers?	N/A
16. Description of sample	What are the important characteristics of the sample? e.g. demographic data, date	N/A
Data collection		
17. Interview guide	Were questions, prompts, guides provided by the authors? Was it pilot tested?	pg.4
18. Repeat interviews	Were repeat inter views carried out? If yes, how many?	N/A
19. Audio/visual recording	Did the research use audio or visual recording to collect the data?	pg.4
20. Field notes	Were field notes made during and/or after the inter view or focus group?	pg.4
21. Duration	What was the duration of the inter views or focus group?	pg.4
22. Data saturation	Was data saturation discussed?	pg.3
23. Transcripts returned	Were transcripts returned to participants for comment and/or correction?	pg.4
**Domain 3: analysis and findings**		
Data analysis		
24. Number of data coders	How many data coders coded the data?	pg.4
25. Description of the coding tree	Did authors provide a description of the coding tree?	N/A
26. Derivation of themes	Were themes identified in advance or derived from the data?	pg.4
27. Software	What software, if applicable, was used to manage the data?	MAXQDA 2018
28. Participant checking	Did participants provide feedback on the findings?	pg.4
Reporting		
29. Quotations presented	Were participant quotations presented to illustrate the themes/findings? Was each quotation identified? e.g. participant number	All throughout Results (pg. 4-9)
30. Data and findings consistent	Was there consistency between the data presented and the findings?	Yes (results/discussion)
31. Clarity of major themes	Were major themes clearly presented in the findings?	Results (pg.4-9)
32. Clarity of minor themes	Is there a description of diverse cases or discussion of minor themes?	pg.5

For peer review only

BMJ Open

Evidence of health system resilience in Myanmar during Cyclone Nargis: perspectives from international organisations

Journal:	*BMJ Open*
Manuscript ID	bmjopen-2021-050700.R1
Article Type:	Original research
Date Submitted by the Author:	11-Aug-2021
Complete List of Authors:	Grimm, Pauline Yongeun; Swiss Tropical and Public Health Institute; University of Basel<br>Merten, Sonja; Swiss Tropical and Public Health Institute; University of Basel<br>Wyss, Kaspar; Swiss Tropical and Public Health Institute; University of Basel
**Primary Subject Heading**:	Global health
Secondary Subject Heading:	Qualitative research, Public health, Health services research, Health policy
Keywords:	QUALITATIVE RESEARCH, PUBLIC HEALTH, Health policy < HEALTH SERVICES ADMINISTRATION & MANAGEMENT

SCHOLARONE™
Manuscripts

I, the Submitting Author has the right to grant and does grant on behalf of all authors of the Work (as defined in the below author licence), an exclusive licence and/or a non-exclusive licence for contributions from authors who are: i) UK Crown employees; ii) where BMJ has agreed a CC-BY licence shall apply, and/or iii) in accordance with the terms applicable for US Federal Government officers or employees acting as part of their official duties; on a worldwide, perpetual, irrevocable, royalty-free basis to BMJ Publishing Group Ltd ("BMJ") its licensees and where the relevant Journal is co-owned by BMJ to the co-owners of the Journal, to publish the Work in this journal and any other BMJ products and to exploit all rights, as set out in our licence.

The Submitting Author accepts and understands that any supply made under these terms is made by BMJ to the Submitting Author unless you are acting as an employee on behalf of your employer or a postgraduate student of an affiliated institution which is paying any applicable article publishing charge ("APC") for Open Access articles. Where the Submitting Author wishes to make the Work available on an Open Access basis (and intends to pay the relevant APC), the terms of reuse of such Open Access shall be governed by a Creative Commons licence – details of these licences and which Creative Commons licence will apply to this Work are set out in our licence referred to above.

Other than as permitted in any relevant BMJ Author's Self Archiving Policies, I confirm this Work has not been accepted for publication elsewhere, is not being considered for publication elsewhere and does not duplicate material already published. I confirm all authors consent to publication of this Work and authorise the granting of this licence.

For peer review only

Evidence of health system resilience in Myanmar during Cyclone Nargis: perspectives from international organisations

Pauline Yongeun Grimm*, Sonja Merten, Kaspar Wyss

***corresponding author: Socinstrasse 57, 4051 Basel, Switzerland / pauline.grimm@unibas.ch**

Affiliations for all authors:

1. **Swiss Tropical and Public Health Institute, Basel, Switzerland**
2. **University of Basel, Basel, Switzerland**

ABSTRACT

Objective
The aim of this study is to improve the understanding of the characteristics of health system resilience in Myanmar's response to Cyclone Nargis and to explore ways to improve resilience at the system level.

Design and Setting
This is an explanatory qualitative study exploring the institutional capacity of resilience in Myanmar's health system. Analysis proceeded using a data-driven thematic analysis closely following the framework method. This process enabled comparisons and contrasts of key emergent themes between the participants, which later generated key results describing the foundational assets, barriers and opportunities for achieving resilience in Myanmar.

Participants
The study comprised of 12 in-depth interviews conducted with representatives from international organisations and non-governmental organisations. The inclusion criteria to recruiting the participants were that they had directly been a part of the Cyclone Nargis response at the time. There was a balanced distribution of participants across UN, bilateral, and NGOs and most of them were either Myanmar citizens or expatriates with extensive working experience based in Myanmar.

Results
Key findings elucidate the characteristics of resilience that have been salient or absent in Myanmar's response to Cyclone Nargis. Strong social capital and motivation propelled by its deep-rooted culture and religion served as Myanmar's greatest assets that filled major gaps in the system. Meanwhile, its post-colonial and military legacy posed barriers towards investing in building its long-term foundations towards resilience.

Conclusions
This study revealed that resilience in the health system can be built through strategic investments towards building the foundations of resilience to better prepare for future shocks. In the case of Myanmar, social capital and motivation, which surfaced as its foundational assets can be channelled into opportunities that can help achieve its long-term health goals, accelerating its journey towards resilience in the health system.

Strengths and limitations of this study

- This study tests the applicability of a conceptual framework for the first time in a lower-middle-income country and builds on the health system resilience conceptual framework developed from a previous systematic review.

For peer review only - http://bmjopen.bmj.com/site/about/guidelines.xhtml

- The topic of health system resilience has a potential to make a timely contribution to the health systems and policy research community, with lessons generated that can be transferred to other contexts.
- The study only represents the view of international organisations and non-governmental organisations, restricting the scope of study to the perceptions of a single stakeholder group, which limits the generalizability of the findings.

INTRODUCTION

Health system resilience

The 2014-15 Ebola epidemic in West Africa served as a turning point in global health. Resilience was introduced as a concept relevant in the discourses for health system strengthening as well as for crisis management in fragile settings (1). Since then, there has been a plethora of literature trying to define, measure, and characterize the concept (2), (3), (4). Resilience is once again receiving more attention than ever with the failure to contain the on-going battle against Covid-19 pandemic.

Health system resilience is defined as the capacity to prepare for and effectively respond to crises whilst retaining core health system functions when a crisis hits. An important aspect concerns the capability of the system to reorganise itself to meet the evolving needs of the situation (5).

Several scholars among many have proposed varying conceptual frameworks in order to elucidate the complex nature of health system resilience. In the aftermath of the Ebola crisis, Kruk et al responded to the growing demands of multilateral organisations to illustrate five key characteristics of a resilient health system and a proposed resilience index corresponding to these five characteristics to measure resilience (6). Blanchet et al proposed a new model of resilience as an underlying management and governance capacity to absorb, adapt and transform itself in case of a shock (7). Gilson et al and Barasa et al introduced the idea of 'everyday resilience,' highlighting the capacities and resources that are required when delivering health services every day, which have to be built over a longer period to address chronic deficiencies in the health system (8), (9). Building on these ideas, Grimm et al have synthesized empirical studies from low- and middle- income countries to confirm Kruk et al's five attributes of resilience and to identify five new characteristics that can serve as foundations to be prioritized in resource-constrained settings to unlock system-level resilience (10). As Grimm et al's five key foundations of resilience are critical inputs to activate Kruk et al's five key attributes of resilience, the paper has employed the five key attributes to develop the interview guide. The analysis applies Grimm et al's framework to test its applicability in Myanmar's specific context.

In addition to the relevant frameworks, the role of the communities is critical when discussing resilience in low- and middle- income countries. A few empirical studies have revealed the vital role community engagement plays in building trust in the health system, which is in turn intricately linked with effective management of crises (11), (12), (13).

Cyclone Nargis and its legacy for Myanmar

A category three Cyclone Nargis struck Myanmar on the 2nd of May, 2008, affecting more than 50 townships and devastating mainly the Ayeyarwady Division and its capital, Yangon. It is recorded as Myanmar's worst natural disasters in recorded history (14). Within this area, the official death toll was estimated 138,000 (15), but the damage had severely affected at least 2.4 million lives in the most affected regions. Not only did the cyclone result in a widespread

For peer review only - http://bmjopen.bmj.com/site/about/guidelines.xhtml

destruction of homes and major infrastructure, but close to 75% of the health facilities were damaged in the affected townships. Most of the destroyed facilities were that of primary health care, which exacerbated healthcare access to the rural poor and amplified economic inequalities (14), (16). TheMyanmar government at the time missed the critical period following the cyclone to implement essential measures to save lives and provide relief efforts (17). The agreement to issue visas for foreign humanitarian aid workers and allow international assistance came three weeks after the storm, only after the UN Secretary-General Ban Ki-moon visited Yangon to plead with General Than Shwe, the head of the military state (18). To this day, the response to Cyclone Nargis reverberates lessons for the international community.

Objectives

The aim of this study is to understand how the characteristics of health system resilience have played out in Myanmar's response to Cyclone Nargis and to explore ways to improve resilience at the system level. Using a refined conceptual framework for health system resilience developed previously, this paper analyses Myanmar's assets and barriers to achieving health system resilience and highlights the lessons learnt from its devastating past with the hope that the lessons may be transferrable to other countries (10).

METHODS

Study setting

This is an explanatory qualitative study exploring the institutional capacity of resilience in Myanmar's health system. Myanmar was recently ranked second on the Global Climate Risk Index of countries most affected by extreme weather events from 1999 to 2018 (19), and 17th out of 191 countries on the Index for Risk Management (INFORM), which assesses the risk of humanitarian crisis and disasters that could overwhelm national capacity to respond (20). It is prone to various natural hazards such as earthquakes, floods, cyclones, droughts, and fires, which have intensified with the effects of climate change (21).

Myanmar's national health plan (2017-2021) highlights Universal Health Coverage (UHC) as its overarching goal and the Ministry of Health and Sports have initiated steps to improve compliance to the International Health Regulations (IHR) in the recent years (22), (23). Initiated by the Union Minister of Health and Sports, Myanmar was the third country in the Southeast Asia region to take part in the Joint External Evaluation (JEE) in 2017 to assess the country's capacity under the IHR to prevent, detect, and respond to public health threats (24). In order to follow-up on the recommendations from the JEE findings, Myanmar's Ministry of Health and Sports have developed a five year National Action Plan for Health Security (NAPHS) (25). These efforts demonstrate the country's political will to embark on a journey towards building the foundations of health system resilience.

Study design and participants

The study comprised of 12 in-depth interviews conducted between January and February 2020, with its participants purposively selected through a snowball sampling of relevant referrals via contacts introduced through the author PYG's existing network whilst working and living in Myanmar (26). This proved as an effective method to track participants that had been directly involved in the response efforts of Cyclone Nargis in 2008. In order to partially offset a possible recall bias due to the time lag between the actual event and data collection, triangulation through supplementary data such as event pictures, reports and media clips were provided by the participants when available, to add to the validity of the data collected. Among the many stakeholder groups, only those who had represented or currently represents

For peer review only - http://bmjopen.bmj.com/site/about/guidelines.xhtml

international organisations and non-governmental organisations have been included in the interviews. There was a balanced distribution of participants across UN, bilateral, and NGOs. Most of them were either Myanmar citizens or expatriates with extensive working experience based in Myanmar. As the interviews targeted a single stakeholder group, the 12 interviews were shown sufficient to reach data saturation (27).

Data collection

The interviews lasted between 1-1.5 hours respectively and were conducted in-person by the author PYG (PhD Candidate) in English. There were no prior exchanges between the participants and the data collector and the interviews were held in private office spaces of the participants. A semi-structured interview guide was developed to explore the roles of the participant, actions taken as an organisation, and perceptions of the strengths and weaknesses in the health system at the time of the crisis. The questions were framed around Kruk et al's five dimensions of resilience (5). See supplementary file for the full interview guide. All interviews have been audio-recorded following informed consent and thereafter transcribed verbatim. Interviewees were approached a second time via video conference in order to verify the accuracy of the transcripts.

Data analysis

Analysis proceeded using a data-driven thematic analysis, closely following the steps of the framework method (28). Verbatim transcriptions and field notes were reviewed for familiarization and verified for accuracy. Following the development of an initial codebook inspired by the ten themes previously developed by the author's team, all transcripts were coded line-by-line using MAXQDA 2018. A hybrid of deductive and inductive approaches provided for a broader structure of the categories and flexibility of codes from the open coding process. Grimm et al's conceptual framework was used to develop the analytical framework (10). The framework was then applied systematically to chart the relevant summary of the transcript with direct quotations structured around 10 categories and 21 codes, resulting in a complete framework matrix. This process enabled comparisons and contrasts of key emergent themes between the participants, which later generated key results describing the foundational assets, barriers and opportunities for achieving resilience in Myanmar. A few of the interviewees provided feedback on the final analysis through a subsequent virtual meeting arranged by the author.

Ethical considerations

The proposal for this study was approved in Myanmar by the Institutional Review Board of the University of Public Health and the Ministry of Health and Sports of the Republic of the Union of Myanmar (2020/Research/4). It has also received clearance in Switzerland by the Ethikkommission Nord-west und Zentralschweiz (Req-2019-00348).

Patient and public involvement

The research was done without public involvement.

RESULTS

The results section presents key findings elucidating the characteristics of resilience that have been salient in Myanmar's response to Cyclone Nargis. The results are categorised into assets, barriers, and future opportunities for achieving health system resilience.

For peer review only - http://bmjopen.bmj.com/site/about/guidelines.xhtml

Foundational assets that can unlock Myanmar's health system resilience

Strong social capital comprised of informal channels and trusted networks

Most participants pointed to the extensive role that the informal sector plays in filling the gaps of the health system. A case in point, the ambulance system in Myanmar is charity-based and subjected to resource availability from various donors. The township hospitals themselves have to seek out appropriate charity groups to maintain the referral system comprised of ambulances and drivers. Likewise, when Cyclone Nargis hit, there were various community-based organisations as first responders of the crisis, offering food, shelter and basic commodities to the affected villages. Strong social capital in Myanmar were exhibited mainly through informal channels, whether it be via monasteries and community-based charity groups. They were able to operate without legal mandates and special memorandum of understanding, precisely because they have obtained people's trust and leveraged the familiarity of their own network. One NGO worker described:

> *"There was an existing social community that was ready to donate to religious events and they were actively involved and participated in the relief efforts after Nargis hit. Each village has a social committee and the religious leaders in the monastry have their own shelters for the affected people who sought refuge. The communities themselves know their needs and gaps the best, which can build resilience." (I09)*

In a context where access to the affected regions were limited for the first three weeks after the cyclone, community engagement proved vital in offering timely support. It was apparent that participants who visited the affected areas experienced severe logistical challenges. Some of the areas were only accessible by boat or in off-road conditions where a robust 4x4 vehicle was required. In this endemically challenging environment, mobile clinics were established to reach the communities in remote areas. All the more in these contexts, community-based resilience was not optional but imperative.

> *"People had to travel long distances to access health care, which severely limited preventative care and people accessing the health system…One thing that consistently stood out to me was the incredible self-sufficiency of the communities themselves, their resilience, and their ability to get back on their feet with the limited resources that they had." (I11)*

Referring back to the example of charity-based ambulances, a few participants stated their concerns that this heavy reliance on informal channels can in turn be a barrier towards building basic systematic preparedness, as often these groups can run without standardized procedures and beget further inequalities in service delivery.

> *A basic preparedness however is not equipped because of this reliance on informal channels. Not all township hospitals have ambulances. Apparently, there is no standard. An ambulance and a driver are not part of a township health department set up. (I06)*

As evidenced through Cyclone Nargis, social capital albeit informal in nature served as an asset for Myanmar in both crisis and calm.

Cultural and religious commitment as a propeller for motivation

Underpinning the strong social capital lay the commitment of community-based groups as the backbone of relief efforts. Many attested that Myanmar's history of prolonged isolation and under-development led to a stronger demonstration of resilience at the community level especially in a situation of emergency. In Myanmar, volunteerism is a common method of civic participation (29). The motivation for this commitment appeared to have stemmed from the people's religious and cultural beliefs to give to charity and donate to the less fortunate, which also manifested in the case of Nargis. A UN staff member shared:

For peer review only - http://bmjopen.bmj.com/site/about/guidelines.xhtml

"People in a community supporting other people in need is a powerful response. Most believe that it's because they were closed off for so long, and it was just helping themselves, and the nature of the general beliefs and teachings of the country. People will go to churches, pagodas, temples, monastries or other public buildings to shelter, and the community there will provide food for a couple of days generally." (I07)

Traditional social welfare support groups revolving often through informal channels played a vital role at the village level and many self-help groups sprung up spontaneously to join the momentum (30). The monasteries even had an informal system to store food collected through donations and shared them with the communities in need. They were the sole sources of aid in the immediate aftermath of Cyclone Nargis until support arrived from the government and UN agencies, which proved as a clear asset. Several interviewers echoed that these social determinants of religion and culture provided a supporting environment for subsequent relief projects to flourish.

"The Buddhist culture compels people to donate to those in need and the monks. The first responses have always come from the people/monasteries, later from the government and finally from the UN agencies. It's because it's part of the culture, all these donations." (I01)

"There are social determinants at play in the response to Nargis. At the time of the disaster, 90% of the people were Buddhist. Those from non-affected townships even came. Came to donate. This is the culture of Buddhism. Supporting environment was religion." (I05)

Community resilience in Myanmar seems to have been built on a stronger foundation because it stems from religion and culture as a motivating force. The powerful source of solidarity within some of the affected communities weathered through generations of political and ethnic divisions.

Barriers to Myanmar's journey towards resilience

Historical legacy affecting transparency, openness and decentralisation

When reflecting upon the aftermath of the cyclone, all participants called out the lack of transparency and openness in how the government had dealt with the crisis at hand. The government at the time did not convey the exact situation analysis to the public, withheld key information about the cyclone, and controlled data such as the degree of damage and casualties incurred. Such lack of transparency not only undermined public trust towards the government but also limited international response teams from accessing Myanmar, which resulted in an overall delay in collective response efforts.

"The government at that time was so quiet and the civil society has not seen the first response from the government. INGOs were not able to access the area until three weeks after the cyclone hit. Perhaps, this is the reason why the government also tried to hide the information as much as possible, for instance, the number of casualties, number of shelters required, etc. We learnt the lack of government information is tied to the lack of accountability." (I10)

Though the reasons may be complex, most respondents pointed to Myanmar's long history of military rule that has operated based on a rigid hierarchy and centralized decision-making system, a context that may not have been conducive for disaster management. There was no proper disaster management mechanism in place; rather, coordination was led through the health cluster. As it had been a highly centralized system, however, the senior general still had the final say and was initially not open to collaborating with the outside world.

"Everything had to be reported and decided by the senior general (Than Swe)..Whatever he said, everybody has to do it." (I04)

For peer review only - http://bmjopen.bmj.com/site/about/guidelines.xhtml

To this day, the Memorandum of Understanding (MoU) is signed at the national level, not with the state. The requests may be made locally, but the final say comes centrally. One participant stated that this hierarchical structure produces dissonant voices at different levels, complicating the decision-making process and posing a risk of not meeting the actual needs on the ground. Another participant echoed this sentiment:

> *"Myanmar is too centralized, which poses a key challenge towards building a resilient health system. Evidence-based planning at the local level is not possible. For example, prevention of the polio outbreak that occurred in Hpa-bun would have been possible if local collaboration with the ethnic organisation could be done at the state level, where states have the decision making power and have fiscal decentralisation." (I06)*

Many participants pointed to theadded layer of complication from the presence of ethnic armed groups in self-administered zones that are run independently of the government as a result of its ongoing strife towards independence (31). These special subnational administrations run by multiple ethnic groups not only have their own health system parallel to that of the Myanmar government but selectively collaborates with local NGOs and community-based groups where there are no conflicts of interest. One participant shared that the polio outbreak in May of 2019 in one of these self-administered regions was particularly difficult to contain due to lack of information shared and restricted access to the area.

> *"Often ethnic health organisations work in these areas to provide services, and some are in fact coordinating well with the ministry. But, access can be difficult, as in the case of the polio outbreak, and the ethnic health organisation wouldn't let the ministry in nor UN agencies. They only allow one NGO they trust to have access. "If you're looking at some kind of outbreak, there is a needed preparedness element by the ministry, but they also need to consider this other factor that is not something that other countries should necessarily worry about." (I06)*

Often UN agencies and NGOs offer to bridge these gaps in services, yet these two parallel systems pose great challenges for international actors to maneuver through the bureaucracies in these conflict zones. It is an ongoing challenge that Myanmar needs to overcome in order to reach an inclusive state of resilience at the national level.

Lack of investments in long-term variables of resilience

Cyclone Nargis revealed the fragility of its health system, especially the areas that had required long-term investments. One participant that had worked with the UN agency during this time depicted how dire the situation had been.

> *"The basic fabrics of the health system were not existing. There was not a place to even fit supplies, medicines and computers. Many RHCs were not functioning well, many times lacking midwives and essential medicine. The nurse to patient ratio was too low and building the nursing infrastructure was critical." (I04)*

According to these first-hand accounts, Myanmar still had a very basic infrastructure, township hospitals covering up to 200,000 people, and a rural health centre serving around 20,000-25,000 people (14). A typical rural health centre had a midwife but this was not necessarily the case in remote and hard-to-reach villages. Health worker shortages came up in all the interviews as a major obstacle for Myanmar's achieving its Universal Health Coverage goal, let alone its journey towards resilience. One UN staff indicated that on average only 54% of the sanctioned health worker posts are filled in Myanmar and building a strong human resource cadre that is evenly distributed across the health system requires a conscious long-term commitment and investment from the government.

> *"Though development cooperation can support buildings, supplies and equipments, robust human resources require a long-term commitment and investment that should come from the government..I*

For peer review only - http://bmjopen.bmj.com/site/about/guidelines.xhtml

mean, the largest issue of Myanmar is that you have only 54% of sanctioned posts filled. Even the 54%, if you see differences in regions." (I06)

All participants stressed the importance of investing in long-term infrastructure, including its health workforce across the country, particularly focusing on extending its health system coverage to remote and vulnerable areas in order to build resilience of the health system as a whole. However, the stark inequalities in remote areas and the frequent staff turnover at all levels of the government were reflections of poor working conditions and weak human resource policies. Participants currently working for a major UN agency shared that funding additional trainings may not be the solution to this health workforce challenge.

"Because of the weakness in the human resource policy, and the working nature of the government job, many government staff are resigning, quitting their jobs. We are losing qualified candidates. Human resource is a major challenge...I know many trainings were supported by organisations and donors, but the problem is the retention of human resource. After the trainings, they move or resign. I think training should not be the key weakness." (I02)

Some specifically referred to the importance of foresight for these long-term commitments. There seems to be a significant gap between plans on paper and implementation as mentioned by one civil society representative:

"The challenge in the state/region is human resource shortages, especially medical doctors. The analysis of the ministry of health national health plan reveals that across the country there is a health service gap due to the lack of human resources. There are not enough medical doctors trained through the medical universities. The government already has a plan, but there is a big implementation gap." (I10)

Myanmar's future for resilience

Harnessing social capital to unleash the power of collective civic duty through civil society groups

As echoed by multiple participants, Myanmar appears to have the ingredients for resilience within its own respective communities. Not only did existing community-based groups act as first responders of Cyclone Nargis albeit decades of dictatorship, but even the coordination took place naturally and provided mutual support. It was simply the people exhibiting civic duty and playing their due role in the society. One representative of a local civil society group shared that health system resilience may be reached when civil society groups build community resilience and start working together with a health system that is also investing in building its strengths.

"At the time of the cyclone, the local civil society groups in each area organised and coordinated with each other by themselves. We haven't seen any government, like red cross, or police. We haven't seen any solider. During the Cyclone Nargis, we have seen the communities, the people, how did they respond. They came out to the street and they supported each other. They tried to remove all the trees and blocks. We learnt from them. We have strong resilient communities. Communities know best what the biggest challenge and needs are in those areas. If we work together, community resilience strengthening and health system strengthening, we could be working towards resilience." (I10)

The informal channels of self-formed community-based groups can be a strong basis for the institutionalization of civil society groups that have a rather complicated relationship with the government. Since Myanmar has opened up in 2011 under President Thein Sein, its reform process has gradually enabled a platform for civil society to establish civil liberties through its '2008 Constitution,' with the article 354 stating the right to freedom of expression, assembly and association (32). Furthermore, the Parliament has enacted the Association Registration Law, which defines and clarifies local and international non-governmental organizations and stipulates requirements to register with the government. These reforms have gradually shifted the landscape of civil society engagements at all levels of the country.

For peer review only - http://bmjopen.bmj.com/site/about/guidelines.xhtml

For peer review only

Even at the national level, the government has been more receptive to the inputs of the civil society groups, including the Ministry of Health and Sports with many of its community-driven development projects. There are certainly growing opportunities for civil society groups to participate in the various development sector working groups (33). One participant reflects on the evolution of civil society engagement since Cyclone Nargis and projects that no crisis can be handled without the involvement of the country's civil society groups.

> *"The health system at that time was already weak and the ministry of health didn't have a strong coordination with the civil society or community groups. The government organised many meetings but had no idea how to work with the local civil society. Only in the last four years did this relationship change. The minister of health stressed the importance of working with CSOs across the country. If an outbreak were to happen, the ministry of health cannot handle the situation without civil societies." (I10)*

Connecting its overarching health goals with long-term investments to improve resilience

As Myanmar is situated in a disaster-prone area, it has naturally developed a local expertise over time through handling its yearly floods. One participant stated that there is an emergency fund stipulated by law to set aside a disaster management fund that can be used to support sub-national levels in case of a potential crisis. How the legal frameworks and plans align with its budgeting and allocation, however, is a different issue.

A recurring theme that transpired when discussing the government's response to the cyclone and other subsequent crises were the emergence of discrepancies between planning, budgeting and implementation. As there has been no clear alignment between its long-term health policy and crisis management, the targeted investment towards building system-level resilience has also lagged behind.

> *"There is very remote connection between planning and budgeting. It happens both at the national and local levels. Preparedness is there on paper." (I06)*

One participant called this the ability to "expand and contract," which also corresponds to the concept of adaptiveness in Kruk et al's framework. It is exhibited by the ability to reallocate its resources to where it is most needed on time and to delegate the authority to the appropriate decentralized administration when necessary (6). It is a vital characteristic of resilience, which is a byproduct of long-term investments and painstaking reforms (34).

> *"This kind of planning, the constant expansion and contraction that you would need in an environment like Myanmar that's so natural disaster heavy, is difficult." (I07)*

Many participants agreed that Myanmar ought to invest towards building long-term infrastructure especially in remote areas and prioritize in its human resource preparedness in order to build resilience in the system for future shocks. This may involve employing creative ways to increase staff retention through the engagement of the private and non-profit sectors. More importantly, however, these plans ought to connect back to its overarching national goal for Universal Health Coverage and its momentum to strengthen its commitment towards International Health Regulations.

> *"In order to improve health system resilience, it is necessary to improve infrastructure needed at the village level… A challenge in building the long-term capacity of health workers, however, is the frequent turnover of staff. Mostly staff assigned for the rural health centres are based there for 1-2 years and move to a better place. Turnover was quite quick." (I09)*

DISCUSSION

For peer review only - http://bmjopen.bmj.com/site/about/guidelines.xhtml

This study explored the degree to which key characteristics of health system resilience manifested in Myanmar's response to Cyclone Nargis and their respective historical and socio-political determinants. As health systems are complex and evolving in nature, it is important to predicate this study on the view that resilience is a capacity that can be nurtured with time and commitment (35). Moreover, facets of resilience should not be seen as disconnected inputs, but rather a holistic result of targeted investments and a series of structural reforms (10), (34).

In the case of Myanmar, it had been the 'everyday resilience' of its frontline workers and communities that had been built through its day-to-day chronic struggles in the context of its weak governance and political and economic vulnerabilities, that in turn prepared them for the unexpected shock (8). As depicted in Figure 1, key themes that emerged from the analysis have been organised into assets, barriers and future opportunities in describing Myanmar's journey towards resilience.

Figure 1. Assets, barriers and opportunities for Myanmar's journey towards health system resilience

First, social capital and motivation have surfaced as key foundational assets in Myanmar that are indicative of its community resilience. The power of collective civic duty filled the service gaps that the government had failed to provide in the immediate aftermath of the cyclone. The social capital present in Myanmar during Cyclone Nargis were comprised of informal channels and trusted networks in the communities, its motivation stemming from a deeper faith rooted in religion and culture. A recent qualitative synthesis highlighted social capital as the interconnecting mechanism common to both health system strengthening and resilient responses to crises, a powerful tool that can unlock resilience of the health system (10). This study extends the notion a step further by demonstrating that the power of social capital can go beyond its health system capacity. Social capital may in fact play a more important role in providing relief from the cyclone, just as it had been reported as being more vital in mitigating the spread of the Ebola virus than any donor-driven short-term interventions (13). The pivotal role social capital plays in building community resilience have been well documented by other studies in the context of both natural and man-made disasters (36), (37), (38), (39), (40). When communities themselves start carrying out critical health system functions, this collective momentum for community resilience prompts what Barker et al calls a 'fortuitous cycle' of increased trust, improved communication and continued meaningful community engagement, all of which are crucial preconditions for enabling health system resilience (11).

Second, Myanmar's historical legacy of prolonged military rule and a lack of long-term investments posed as major barriers to achieving resilience. The lack of transparency and openness stood out as the biggest obstacle towards crisis management, which undermined public trust towards the government and hampered international response teams from accessing Myanmar in the initial stages. Myanmar is a prime example of a post-colonial state which development experts describes as a 'conflict trap' (41). The legacy of the colonial experience, ethnic insurgencies, and a successive military rule, shifted all of its government's priorities to regaining its national sovereignty and ensuring security through increased military spending (42). Consequently, little had been left for health and social welfare, with only approximately 2% of its GDP spent on health care shortly before the cyclone hit (30). It is no surprise that Myanmar's health system was fragile and not ready to handle a disaster of such magnitude.

Governance practices influence organizational resilience and decentralized governments are certainly at an advantage to exhibit the necessary flexibility and timely responses required in

For peer review only - http://bmjopen.bmj.com/site/about/guidelines.xhtml

crisis management (2). Having a decentralized governance structure itself, however, does not guarantee a successful response. China had no national disaster management agency nor related policies during the Sichuan earthquake, but effectively controlled the emergency with its President's charismatic leadership and vertical administrative structure (43). Myanmar's centralized and hierarchical system could have been used for Myanmar's advantage, but an adequate disaster management had not been the priority of the government.

A common pattern across failed attempts in responding to a crisis was to prioritize on the 'fast variables,' delivering services in the form of temporary shelters and supplies without considering its longer-term infrastructure building, as it had been in the case of Liberia during its Ebola crisis (44). Building resilience, however, is much more than preparedness, as Kruk et al states, that it requires investing in systems and its corresponding 'slow variables' that can function in both crisis and calm (6). A health system that has a clear foresight for its people will strengthen its health system infrastructure and train its human resource cadre to build the foundations of its health system (10). In the context of Myanmar, it would mean to apply a systems thinking approach in examining its past shortcomings and redesigning its institutions and organisational structures to serve its evolving purpose and prepare for future shocks (45).

Third, Myanmar may channel its assets into opportunities by using its growing civil society platforms and leveraging its long-term national health goals. Cyclone Nargis revealed that Myanmar has the key ingredients for resilience within its own communities through its people exhibiting their civic duty. Civil society has had a long history in Myanmar dating back to its informal Buddhist associations in the traditional kingdoms and various other religious groups organizing social and welfare programmes during its colonial period and beyond (46). Since Cyclone Nargis, the space for civil societies further expanded through its legal reforms but at the end of the day only a few have been able to obtain official registration and secure a sustainable funding mechanism (46). According to the Civil Society Survey conducted in 2019, despite its marked progress, a wide range of obstacles still remain and hinder civil societies from reaching their goals (32). Myanmar has set ambitious national goals to reach Universal Health Coverage (UHC) and to improve compliance to the International Health Regulations (IHR) in order to ensure healthcare and health security for all (22), (25). As evident in its neighbouring countries, however, civil society engagement and community feedback loops for policy and decision-making processes have shown as prerequisites to accelerate progress towards achieving these compendium of goals (47). Trust and accountability at all levels in the system are what builds the 'capacity of legitimacy,' a crucial component of resilient health systems, that can only be strengthened through an inclusive consultation process of engaging communities and civil society groups (7). Myanmar has to realize that strategically leveraging these opportunities may be the fastest route to building resilience.

The findings of this study offer tangible suggestions for Myanmar to invest on building its hard and soft long-term infrastructures in remote areas, prioritising in human resource preparedness, and redesigning institutions and organisational structures to serve its evolving purposes and to prepare for future shocks.

Study Strengths and Limitations

This study is empirical in nature, building on a health system resilience conceptual framework developed from a previous systematic review. The framework has been tested for the first time for its applicability in a lower-middle-income country. Regarding the study design, the long time lag between the actual event and data collection introduces a potential recall bias, which was partially offset by triangulation through supplementary data such as reports, event

For peer review only - http://bmjopen.bmj.com/site/about/guidelines.xhtml

pictures and media clips provided by the participants. The topic of health system resilience has a potential to make a timely contribution to the health systems and policy research community, with lessons generated that can be transferrable to other contexts. Due to Covid-19 pandemic's travel restrictions imposed during the data collection period, the study only represents the view of international organisations and non-governmental organisations, restricting the scope of the study to the perceptions of a single stakeholder group. Further studies should explore capturing a wider range of stakeholders, the perceptions of communities, frontline workers and government staff that may be able to offer a more holistic view of the phenomenon.

CONCLUSION

This study revealed that resilience in the health system can be built through targeted investments towards building the foundations of resilience to better prepare for future shocks. In the case of Myanmar, social capital and motivation, which surfaced as its foundational assets, may be channelled into opportunities that can help achieve its long-term health goals, accelerating its journey towards resilience in the health system.

ACKNOWLEDGMENTS

The authors would like to extend appreciations to all the interviewees who carved out time to part take in this study. Special thanks to Dr. Nilar Tin for her valuable input on the initial study concept and for suggesting relevant interviewees.

CONTRIBUTORS

PYG and KW conceptualized the study; PYG collected and analysed the data with substantive input from SM and KW. PYG developed the first draft of the manuscript and all authors contributed to the subsequent and final drafts.

FUNDING

This article is part of the PhD project that has received funding from the European Union's Horizon 2020 research and innovation programme under the Marie Skłodowska-Curie grant agreement No. 801076, through the SSPH+ *Global PhD Fellowship Programme in Public Health Sciences (GlobalP3HS) of the Swiss School of Public Health*. The corresponding author is also a recipient of the Swiss Government Excellence Scholarship (ESKAS), provided by the Swiss Federal Commission for Scholarships for Foreign Students (FCS) with the award number 2018.0217. The funding bodies did not play a role in the design, analysis, and writing of the manuscript.

COMPETING INTERESTS

None declared

PATIENT CONSENT FOR PUBLICATION

Not required

ETHICS APPROVAL

The proposal for this study was approved in Myanmar by the Institutional Review Board of the University of Public Health and the Ministry of Health and Sports of the Republic of the Union of Myanmar (2020/Research/4). It has also received clearance in Switzerland by the Ethikkommission Nord-west und Zentralschweiz (Req-2019-00348).

For peer review only

For peer review only - http://bmjopen.bmj.com/site/about/guidelines.xhtml

DATA AVAILABILITY STATEMENT

This is a qualitative study of a small number of interviewees representing international organizations and NGOs. All data relevant to the study are included in the article or as supplementary information. Making the full data set publicly available could potentially breach the privacy commitment made to the participants upon obtaining informed consent, as well as the ethics approval granted by both the Institutional Review Board of the University of Public Health in Myanmar and Ethikkommission Nord-west und Zentralschweiz in Switzerland. Therefore, the authors will not make the full transcripts available to a wider audience.

REFERENCES

1. Global HS. Vancouver statement for the fourth global symposium on health systems research. 2016.
2. Barasa E, Mbau R, Gilson L. What Is Resilience and How Can It Be Nurtured? A Systematic Review of Empirical Literature on Organizational Resilience. International Journal of Health Policy and Management. 2018;7(6):491-503.
3. Fridell M, Edwin S, Von Schreeb J, Saulnier DD. Health system resilience: what are we talking about? A scoping review mapping characteristics and keywords. International Journal of Health Policy and Management. 2020;9(1):6.
4. Biddle L, Wahedi K, Bozorgmehr K. Health system resilience: a literature review of empirical research. Health Policy Plan. 2020;35(8).
5. Kruk ME, Myers M, Varpilah ST, Dahn BT. What is a resilient health system? Lessons from Ebola. The Lancet. 2015;385(9980):1910-2.
6. Kruk ME, Ling EJ, Bitton A, Cammett M, Cavanaugh K, Chopra M, et al. Building resilient health systems: a proposal for a resilience index. BMJ. 2017:j2323.
7. Blanchet K, Nam SL, Ramalingam B, Pozo-Martin F. Governance and Capacity to Manage Resilience of Health Systems: Towards a New Conceptual Framework. Int J Health Policy Manag. 2017;6(8):431-5.
8. Gilson L, Barasa E, Nxumalo N, Cleary S, Goudge J, Molyneux S, et al. Everyday resilience in district health systems: Emerging insights from the front lines in Kenya and South Africa. BMJ Global Health. 2017;2.
9. Barasa EW, Cloete K, Gilson L. From bouncing back, to nurturing emergence: reframing the concept of resilience in health systems strengthening. Health Policy Plan. 2017;32:91-4.
10. Grimm PY, Oliver S, Merten S, Han WW, Wyss K. Enhancing the Understanding of Resilience in Health Systems of Low- and Middle-income Countries: A Qualitative Evidence Synthesis International Journal of Health Policy and Management. 2021:-.
11. Barker KM, Ling EJ, Fallah M, VanDeBogert B, Kodl Y, Macauley RJ, et al. Community engagement for health system resilience: evidence from Liberia's Ebola epidemic. 2020;35(4):416-23.
12. Bhandari S, Alonge OJHrp, systems. Measuring the resilience of health systems in low-and middle-income countries: a focus on community resilience. 2020;18(1):1-19.
13. Alonge O, Sonkarlay S, Gwaikolo W, Fahim C, Cooper J, Peters DJGha. Understanding the role of community resilience in addressing the Ebola virus disease epidemic in Liberia: a qualitative study (community resilience in Liberia). 2019;12(1):1662682.
14. Group TC. Post-Nargis Joint Assessment. 2008.
15. EM-DAT: The Emergency Events Database [Internet]. Universite catholique de Louvain (UCL). Available from: www.emdat.be.
16. Warr P, Aung LLJWD. Poverty and inequality impact of a natural disaster: Myanmar's 2008 cyclone Nargis. 2019;122:446-61.

For peer review only - http://bmjopen.bmj.com/site/about/guidelines.xhtml

17. Stover E, Vinck PJJ. Cyclone Nargis and the politics of relief and reconstruction aid in Burma (Myanmar). 2008;300(6):729-31.
18. Secretary-General's press conference at Hotel Sedona in Yangon [press release]. 23 May 2008.
19. David Eckstein VK, Laura Schäfer, Maik Winges. Global climate risk index 2020 Germanwatch; 2019.
20. INFORM Global Risk Index 2020 [Internet]. European Commission 2019. Available from: https://drmkc.jrc.ec.europa.eu/inform-index/.
21. UNOCHA. HCT Myanmar- Inter-agency emergency response preparedness (ERP) plan 2017.
22. Ministry of H, Sports tRotUoM. Myanmar National Health Plan 2017-2021. 2016.
23. Pyone T, Aung TT, Endericks T, Myint NW, Inamdar L, Collins S, et al. Health system governance in strengthening International Health Regulations (IHR) compliance in Myanmar. 2020;5(11):e003566.
24. Joint external evaluation of IHR core capacities of The Republic of the Union of Myanmar: executive summary. . World Health Organization; 2017
25. National Action Plan for Health Security (NAPHS) 2018-2022. Ministry of Health and Sports, the Republic of the Union of Myanmar 2018.
26. Naderifar M, Goli H, Ghaljaie FJSiDoME. Snowball sampling: A purposeful method of sampling in qualitative research. 2017;14(3):1-6.
27. Guest G, Bunce A, Johnson L. How Many Interviews Are Enough?: An Experiment with Data Saturation and Variability. Field Methods. 2006;18(1):59-82.
28. Gale NK, Heath G, Cameron E, Rashid S, Redwood S. Using the framework method for the analysis of qualitative data in multi-disciplinary health research. BMC Medical Research Methodology. 2013;13(1).
29. Dove CJAJoS-EAS. Giving Trends in Myanmar: More Than Merit Making. 2017;10(2):205-22.
30. The Republic of the Union of Myanmar Health System Review: World Health Organization; 2015 2015.
31. Jolliffe KJTAF. Ethnic armed conflict and territorial administration in Myanmar. 2015:89.
32. Buschmann A, Soe AJAaS. Authoritarian Impediments to Civil Society in Contemporary Myanmar: Findings from the Myanmar Civil Society Survey 2019. 2020.
33. Civil society briefs: Myanmar. Asian Development Bank 2015.
34. Kutzin J, Sparkes SPJBotWHO. Health systems strengthening, universal health coverage, health security and resilience. 2016;94(1):2.
35. Topp SM. Power and politics: the case for linking resilience to health system governance. BMJ Specialist Journals; 2020.
36. Bianca RJTRoAS. Influence of social capital on community resilience in the case of emergency situations in Romania. 2018;14(54):73-89.
37. Aldrich DP, Meyer MA. Social Capital and Community Resilience. American Behavioral Scientist. 2015;59:254-69.
38. Pfefferbaum B, Van Horn RL, Pfefferbaum RLJCSWJ. A conceptual framework to enhance community resilience using social capital. 2017;45(2):102-10.
39. Aldrich DP. The importance of social capital in building community resilience. Rethinking resilience, adaptation and transformation in a time of change: Springer; 2017. p. 357-64.
40. Grimm PY, Saulnier DD. Civic Duty: A Booster for Resilience? International Journal of Public Health. 2021;66.
41. Smith M. State of strife: The dynamics of ethnic conflict in Burma: Institute of Southeast Asian Studies; 2007.
42. Than TMM. Myanmar: preoccupation with regime survival, national unity, and stability. Standford: Stanford University Press; 1998. 390-416 p.
43. Lin L, Ashkenazi I, Dorn BC, Savoia E. The public health system response to the 2008 Sichuan province earthquake: a literature review and interviews. Disasters. 2014;38(4):753-73.

For peer review only - http://bmjopen.bmj.com/site/about/guidelines.xhtml

For peer review only

44. Ling EJ, Larson E, Macauley RJ, Kodl Y, VanDeBogert B, Baawo S, et al. Beyond the crisis: did the Ebola epidemic improve resilience of Liberia's health system? Health Policy Plan. 2017;32(suppl_3):iii40-iii7.
45. Arnold RD, Wade JPJPCS. A definition of systems thinking: A systems approach. 2015;44(2015):669-78.
46. Kramer T. Civil society gaining ground: Opportunities for change and development in Burma: Transnational Institute Amsterdam; 2011.
47. Yeoh E-K, Johnston C, Chau PYK, Kiang N, Tin P, Tang JJHS, et al. Governance functions to accelerate progress toward universal health coverage (UHC) in the Asia-Pacific Region. 2019;5(1):48-58.

For peer review only

For peer review only - http://bmjopen.bmj.com/site/about/guidelines.xhtml

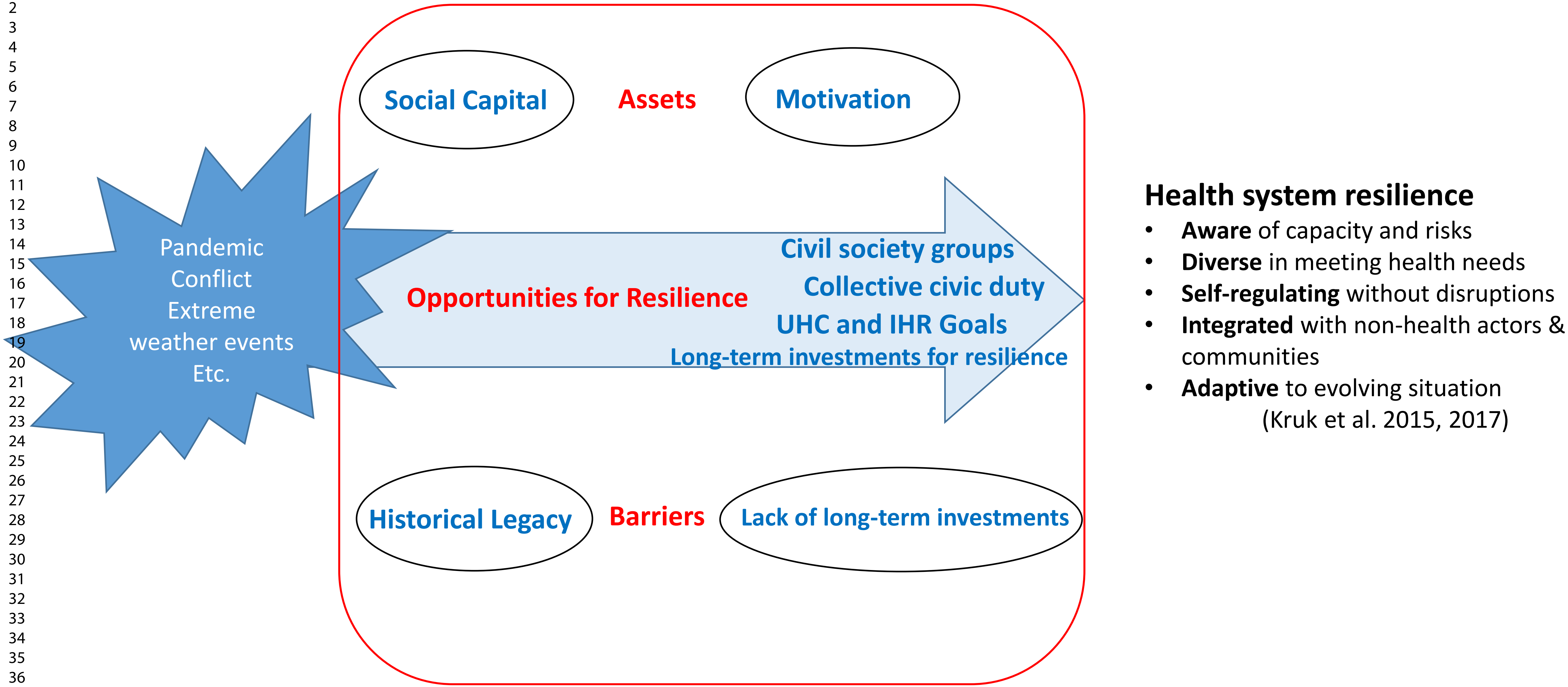

Figure 1. Assets, barriers and opportunities for Myanmar's journey towards health system resilience

For peer review only - http://bmjopen.bmj.com/site/about/guidelines.xhtml

SUPPLEMENTARY FILE. INTERVIEW GUIDE

Study Title: Health System Resilience: key for unlocking the complex health system strengthening agenda

Investigator: ________________________ **Phone number:**___________________________

Organisation/Department: ______________________________ **Date: |__|__|__|__|__|__|**

Position/Title: ____

Stakeholder Group:

□ Central Government

□ Local Government (Township, local health committees, member of parliament, etc)

□ Health workers

□ Int'l Agencies (UN, NGOs, Bilateral, etc)

Number of participants: |__|__|

DRAFT SEMI-STRUCTURED IN-DEPTH INTERVIEW QUESTIONS

Introduction: purpose of research, use of audio tape recorder, confidentiality

I. PARTICIPANT INTRODUCTION

1. What is your role when the country is hit with an external shock, whether it be a natural disaster, unforeseen pandemic, etc.

Prompt: examples of disaster management, pandemic, natural disasters

II. CASE 1: CYCLONE NARGIS (2008)

2. (General) Can you please describe in detail the account of Cyclone Nargis?

Probe: What was your title/position at the time?

What was the response protocol (written or perceived norm)?

What actions (out of the norm) did you take?

How did you respond as an individual?

3. (General) Can you please describe in detail the response actions taken as an organisation that you were a part of at the time?

Probe: Decision-making protocol? Evidence?

For peer review only

4. (General) Can you comment on one strength and weakness that the health system exhibited during Cyclone Nargis?

Probe: Which key element was present or missing during the incident?

5. (Dimension 1_Aware) At the time of Cyclone Nargis, how was the health system monitoring and tracking its health risks?

6. (Dimension 1_Aware) Once the disaster hit, who was included in the communication channel?

7. (Dimension 1_Aware) Who made the decisions regarding resource allocation and coordination efforts?

8. (Dimension 2_Diverse) At the time of Cyclone Nargis, were a range of health services still offered in health facilities (business as usual) or was the quality/quantity of services compromised? Why?

9. (Dimension 3_Self-regulating) Can you recall the extent of non-state organisations intervening to provide support at the time of crisis? (e.g. NGOs, Int'l Orgnisations, private actors, etc)

10. (Dimension 3_Self-regulating) At national level, were there reserve capacity (budget, HR, other resources) to summon quickly?

11. (Dimension 4_Integrated) Can you comment on the level of coordination between government, global and private actors at the time of the crisis?

12. (Dimension 4_Integrated) Who was the main actor responsible for coordination?

13. (Dimension 5_Adaptive) Was there at the time flexibility when it came to spending budget to respond to the emergency?

14. (Dimension 5_Adaptive) Can you comment on how much decision-making was possible at the decentralised/local level?

15. What was the main lessons learnt after undergoing Cyclone Nargis?

III. CONCEPT OF HEALTH SYSTEM RESILIENCE (HSR) AND GENERAL QUESTIONS

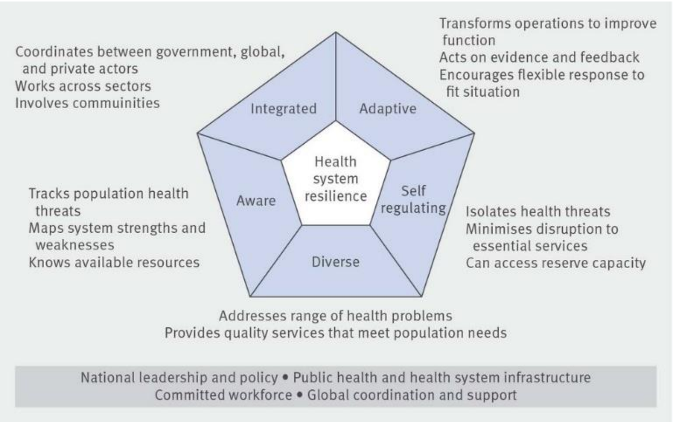

Figure 1. Resilient Health System Framework

For peer review only

16. Reviewing the framework above, how would you describe Myanmar's health system resilience generally?

Probe: Can you give concrete examples that indicate Myanmar's HSR?

17. What is Myanmar's strength/weakness when it comes to resilience?

18. What do you think is the most important element of resilience?

IV. PREPAREDNESS AND FUTURE SHOCKS

19. What may be the biggest threat/risk for Myanmar?

20. At this point in time, how would you describe Myanmar's preparedness for future shocks?

21. What national policies are in place to enable the Ministry of Health to be resilient to future shocks?

22. Can you comment on the efforts taken by the Ministry of Health and Sports to improve preparedness? (HR allocation, crisis management team, emergency plan, trainings undertaken, protocols, etc)

23. What would you advise to improve health system resilience in Myanmar?

Thank you for your time and heartfelt responses to the interview questions.

For peer review only

Consolidated criteria for reporting qualitative studies (COREQ): 32-item checklist

Developed from:
Tong A, Sainsbury P, Craig J. Consolidated criteria for reporting qualitative research (COREQ): a 32-item checklist for interviews and focus groups. *International Journal for Quality in Health Care*. 2007. Volume 19, Number 6: pp. 349 – 357

No. Item	Guide questions/description	Reported on Page #
**Domain 1: Research team and reflexivity**		
Personal Characteristics		
1. Inter viewer/facilitator	Which author/s conducted the inter view or focus group?	pg.4
2. Credentials	What were the researcher's credentials? E.g. PhD, MD	pg.4
3. Occupation	What was their occupation at the time of the study?	pg.4
4. Gender	Was the researcher male or female?	N/A
5. Experience and training	What experience or training did the researcher have?	pg.4
Relationship with participants		
6. Relationship established	Was a relationship established prior to study commencement?	pg.4
7. Participant knowledge of the interviewer	What did the participants know about the researcher? e.g. personal goals, reasons for doing the research	pg.4
8. Interviewer characteristics	What characteristics were reported about the inter viewer/facilitator? e.g. Bias, assumptions, reasons and interests in the research topic	pg.4
**Domain 2: study design**		
Theoretical framework		
9. Methodological orientation and Theory	What methodological orientation was stated to underpin the study? e.g. grounded theory, discourse analysis, ethnography, phenomenology, content analysis	pg.4
Participant selection		
10. Sampling	How were participants selected? e.g. purposive, convenience, consecutive, snowball	pg.3
11. Method of approach	How were participants approached? e.g. face-to-face, telephone, mail, email	pg.4
12. Sample size	How many participants were in the study?	pg.3
13. Non-participation	How many people refused to participate or dropped out? Reasons?	N/A

For peer review only

Setting		
14. Setting of data collection	Where was the data collected? e.g. home, clinic, workplace	pg.4
15. Presence of non-participants	Was anyone else present besides the participants and researchers?	N/A
16. Description of sample	What are the important characteristics of the sample? e.g. demographic data, date	N/A
Data collection		
17. Interview guide	Were questions, prompts, guides provided by the authors? Was it pilot tested?	pg.4
18. Repeat interviews	Were repeat inter views carried out? If yes, how many?	N/A
19. Audio/visual recording	Did the research use audio or visual recording to collect the data?	pg.4
20. Field notes	Were field notes made during and/or after the inter view or focus group?	pg.4
21. Duration	What was the duration of the inter views or focus group?	pg.4
22. Data saturation	Was data saturation discussed?	pg.3
23. Transcripts returned	Were transcripts returned to participants for comment and/or correction?	pg.4
**Domain 3: analysis and findings**		
Data analysis		
24. Number of data coders	How many data coders coded the data?	pg.4
25. Description of the coding tree	Did authors provide a description of the coding tree?	N/A
26. Derivation of themes	Were themes identified in advance or derived from the data?	pg.4
27. Software	What software, if applicable, was used to manage the data?	MAXQDA 2018
28. Participant checking	Did participants provide feedback on the findings?	pg.4
Reporting		
29. Quotations presented	Were participant quotations presented to illustrate the themes/findings? Was each quotation identified? e.g. participant number	All throughout Results (pg. 4-9)
30. Data and findings consistent	Was there consistency between the data presented and the findings?	Yes (results/discussion)
31. Clarity of major themes	Were major themes clearly presented in the findings?	Results (pg.4-9)
32. Clarity of minor themes	Is there a description of diverse cases or discussion of minor themes?	pg.5

For peer review only

BMJ Open

Evidence of health system resilience in Myanmar during Cyclone Nargis: a qualitative analysis

Journal:	*BMJ Open*
Manuscript ID	bmjopen-2021-050700.R2
Article Type:	Original research
Date Submitted by the Author:	06-Sep-2021
Complete List of Authors:	Grimm, Pauline Yongeun; Swiss Tropical and Public Health Institute; University of Basel<br>Merten, Sonja; Swiss Tropical and Public Health Institute; University of Basel<br>Wyss, Kaspar; Swiss Tropical and Public Health Institute; University of Basel
**Primary Subject Heading**:	Global health
Secondary Subject Heading:	Qualitative research, Public health, Health services research, Health policy
Keywords:	QUALITATIVE RESEARCH, PUBLIC HEALTH, Health policy < HEALTH SERVICES ADMINISTRATION & MANAGEMENT

SCHOLARONE™
Manuscripts

I, the Submitting Author has the right to grant and does grant on behalf of all authors of the Work (as defined in the below author licence), an exclusive licence and/or a non-exclusive licence for contributions from authors who are: i) UK Crown employees; ii) where BMJ has agreed a CC-BY licence shall apply, and/or iii) in accordance with the terms applicable for US Federal Government officers or employees acting as part of their official duties; on a worldwide, perpetual, irrevocable, royalty-free basis to BMJ Publishing Group Ltd ("BMJ") its licensees and where the relevant Journal is co-owned by BMJ to the co-owners of the Journal, to publish the Work in this journal and any other BMJ products and to exploit all rights, as set out in our licence.

The Submitting Author accepts and understands that any supply made under these terms is made by BMJ to the Submitting Author unless you are acting as an employee on behalf of your employer or a postgraduate student of an affiliated institution which is paying any applicable article publishing charge ("APC") for Open Access articles. Where the Submitting Author wishes to make the Work available on an Open Access basis (and intends to pay the relevant APC), the terms of reuse of such Open Access shall be governed by a Creative Commons licence – details of these licences and which Creative Commons licence will apply to this Work are set out in our licence referred to above.

Other than as permitted in any relevant BMJ Author's Self Archiving Policies, I confirm this Work has not been accepted for publication elsewhere, is not being considered for publication elsewhere and does not duplicate material already published. I confirm all authors consent to publication of this Work and authorise the granting of this licence.

For peer review only

Evidence of health system resilience in Myanmar during Cyclone Nargis: a qualitative analysis

Pauline Yongeun Grimm*, Sonja Merten, Kaspar Wyss

***corresponding author: Socinstrasse 57, 4051 Basel, Switzerland / pauline.grimm@unibas.ch**

Affiliations for all authors:

1. **Swiss Tropical and Public Health Institute, Basel, Switzerland**
2. **University of Basel, Basel, Switzerland**

For peer review only

ABSTRACT

Objective

The aim of this study is to improve the understanding of the characteristics of health system resilience in Myanmar's response to Cyclone Nargis and to explore ways to improve resilience at the system level.

Design and Setting

This is an explanatory qualitative study exploring the institutional capacity of resilience in Myanmar's health system. Analysis proceeded using a data-driven thematic analysis closely following the framework method. This process enabled comparisons and contrasts of key emergent themes between the participants, which later generated key results describing the foundational assets, barriers and opportunities for achieving resilience in Myanmar.

Participants

The study comprised of 12 in-depth interviews conducted with representatives from international organisations and non-governmental organisations. The inclusion criteria to recruiting the participants were that they had directly been a part of the Cyclone Nargis response at the time. There was a balanced distribution of participants across UN, bilateral, and NGOs and most of them were either Myanmar citizens or expatriates with extensive working experience based in Myanmar.

Results

Key findings elucidate the characteristics of resilience that have been salient or absent in Myanmar's response to Cyclone Nargis. Strong social capital and motivation propelled by its deep-rooted culture and religion served as Myanmar's greatest assets that filled major gaps in the system. Meanwhile, its post-colonial and military legacy posed barriers towards investing in building its long-term foundations towards resilience.

Conclusions

This study revealed that resilience in the health system can be built through strategic investments towards building the foundations of resilience to better prepare for future shocks. In the case of Myanmar, social capital and motivation, which surfaced as its foundational assets can be channelled into opportunities that can help achieve its long-term health goals, accelerating its journey towards resilience in the health system.

Strengths and limitations of this study

- This study tests the applicability of a conceptual framework for the first time in a lower-middle-income country and builds on the health system resilience conceptual framework developed from a previous systematic review.

For peer review only - http://bmjopen.bmj.com/site/about/guidelines.xhtml

- The topic of health system resilience has a potential to make a timely contribution to the health systems and policy research community, with lessons generated that can be transferred to other contexts.
- The study only represents the view of international organisations and non-governmental organisations, restricting the scope of study to the perceptions of a single stakeholder group, which limits the generalizability of the findings.

INTRODUCTION

Health system resilience

The 2014-15 Ebola epidemic in West Africa served as a turning point in global health. Resilience was introduced as a concept relevant in the discourses for health system strengthening as well as for crisis management in fragile settings (1). Since then, there has been a plethora of literature trying to define, measure, and characterize the concept (2), (3), (4). Resilience is once again receiving more attention than ever with the failure to contain the on-going battle against Covid-19 pandemic.

Health system resilience is defined as the capacity to prepare for and effectively respond to crises whilst retaining core health system functions when a crisis hits. An important aspect concerns the capability of the system to reorganise itself to meet the evolving needs of the situation (5).

Several scholars among many have proposed varying conceptual frameworks in order to elucidate the complex nature of health system resilience. In the aftermath of the Ebola crisis, Kruk et al responded to the growing demands of multilateral organisations to illustrate five key characteristics of a resilient health system and a proposed resilience index corresponding to these five characteristics to measure resilience (6). Blanchet et al proposed a new model of resilience as an underlying management and governance capacity to absorb, adapt and transform itself in case of a shock (7). Gilson et al and Barasa et al introduced the idea of 'everyday resilience,' highlighting the capacities and resources that are required when delivering health services every day, which have to be built over a longer period to address chronic deficiencies in the health system (8), (9). Building on these ideas, Grimm et al have synthesized empirical studies from low- and middle- income countries to confirm Kruk et al's five attributes of resilience and to identify five new characteristics that can serve as foundations to be prioritized in resource-constrained settings to unlock system-level resilience (10). As Grimm et al's five key foundations of resilience are critical inputs to activate Kruk et al's five key attributes of resilience, the paper has employed the five key attributes to develop the interview guide. The analysis applies Grimm et al. 2021's framework composed of ten characteristics of resilience to test its applicability in Myanmar's specific context.

In addition to the relevant frameworks, the role of the communities is critical when discussing resilience in low- and middle- income countries. A few empirical studies have revealed the vital role community engagement plays in building trust in the health system, which is in turn intricately linked with effective management of crises (11), (12), (13).

Cyclone Nargis and its legacy for Myanmar

A category three Cyclone Nargis struck Myanmar on the 2nd of May, 2008, affecting more than 50 townships and devastating mainly the Ayeyarwady Division and its capital, Yangon. It is recorded as Myanmar's worst natural disasters in recorded history (14). Within this area, the official death toll was estimated 138,000 (15), but the damage had severely affected at least 2.4 million lives in the most affected regions. Not only did the cyclone result in a widespread

For peer review only - http://bmjopen.bmj.com/site/about/guidelines.xhtml

destruction of homes and major infrastructure, but close to 75% of the health facilities were damaged in the affected townships. Most of the destroyed facilities were that of primary health care, which exacerbated healthcare access to the rural poor and amplified economic inequalities (14), (16). TheMyanmar government at the time missed the critical period following the cyclone to implement essential measures to save lives and provide relief efforts (17). The agreement to issue visas for foreign humanitarian aid workers and allow international assistance came three weeks after the storm, only after the UN Secretary-General Ban Ki-moon visited Yangon to plead with General Than Shwe, the head of the military state (18). To this day, the response to Cyclone Nargis reverberates lessons for the international community.

Objectives

The aim of this study is to understand how the characteristics of health system resilience have played out in Myanmar's response to Cyclone Nargis and to explore ways to improve resilience at the system level. Using a refined conceptual framework for health system resilience developed previously, this paper analyses Myanmar's assets and barriers to achieving health system resilience and highlights the lessons learnt from its devastating past with the hope that the lessons may be transferrable to other countries (10).

METHODS

Study setting

This is an explanatory qualitative study exploring the organisational capacity of resilience in Myanmar's health system. Myanmar was recently ranked second on the Global Climate Risk Index of countries most affected by extreme weather events from 1999 to 2018 (19), and 17th out of 191 countries on the Index for Risk Management (INFORM), which assesses the risk of humanitarian crisis and disasters that could overwhelm national capacity to respond (20). It is prone to various natural hazards such as earthquakes, floods, cyclones, droughts, and fires, which have intensified with the effects of climate change (21).

Myanmar's national health plan (2017-2021) highlights Universal Health Coverage (UHC) as its overarching goal and the Ministry of Health and Sports have initiated steps to improve compliance to the International Health Regulations (IHR) in the recent years (22), (23). Initiated by the Union Minister of Health and Sports, Myanmar was the third country in the Southeast Asia region to take part in the Joint External Evaluation (JEE) in 2017 to assess the country's capacity under the IHR to prevent, detect, and respond to public health threats (24). In order to follow-up on the recommendations from the JEE findings, Myanmar's Ministry of Health and Sports have developed a five year National Action Plan for Health Security (NAPHS) (25). These efforts demonstrate the country's political will to embark on a journey towards building the foundations of health system resilience.

Study design and participants

The study comprised of 12 in-depth interviews conducted between January and February 2020, with its participants purposively selected through a snowball sampling of relevant referrals via contacts introduced through the author PYG's existing network whilst working and living in Myanmar (26). This proved as an effective method to track participants that had been directly involved in the response efforts of Cyclone Nargis in 2008. In order to partially offset a possible recall bias due to the time lag between the actual event and data collection, triangulation through supplementary data such as event pictures, reports and media clips were provided by the participants when available, to add to the validity of the data collected. Among the many stakeholder groups, only those who had represented or currently represents

For peer review only - http://bmjopen.bmj.com/site/about/guidelines.xhtml

international organisations and non-governmental organisations have been included in the interviews. There was a balanced distribution of participants across affiliations and nationalities. There were five UN staff, three bilateral representatives, three NGO workers, six Myanmar citizens and six expatriates. As the interviews targeted a single stakeholder group, the 12 interviews were shown sufficient to reach data saturation (27).

Data collection

The interviews lasted between 1-1.5 hours respectively and were conducted in-person by the author PYG (PhD Candidate) in English. There were no prior exchanges between the participants and the data collector and the interviews were held in private office spaces of the participants. A semi-structured interview guide was developed to explore the roles of the participant, actions taken as an organisation, and perceptions of the strengths and weaknesses in the health system at the time of the crisis. The questions were framed around Kruk et al's five dimensions of resilience (5). See supplementary file for the full interview guide. All interviews have been audio-recorded following informed consent and thereafter transcribed verbatim. Interviewees were approached a second time via video conference in order to verify the accuracy of the transcripts.

Data analysis

Analysis proceeded using a data-driven thematic analysis, closely following the steps of the framework method (28). Verbatim transcriptions and field notes were reviewed for familiarization and verified for accuracy. Following the development of an initial codebook inspired by the ten themes previously developed by the author's team, all transcripts were coded line-by-line using MAXQDA 2018. A hybrid of deductive and inductive approaches provided for a broader structure of the categories and flexibility of codes from the open coding process. As illustrated in Figure 1, Grimm et al's conceptual framework was used to develop the analytical framework (10). The framework was then applied systematically to chart the relevant summary of the transcript with direct quotations structured around 10 categories and 21 codes, resulting in a complete framework matrix. This process enabled comparisons and contrasts of key emergent themes between the participants, which later generated key results describing the foundational assets, barriers and opportunities for achieving resilience in Myanmar. A few of the interviewees provided feedback on the final analysis through a subsequent virtual meeting arranged by the author.

Ethical considerations

The proposal for this study was approved in Myanmar by the Institutional Review Board of the University of Public Health and the Ministry of Health and Sports of the Republic of the Union of Myanmar (2020/Research/4). It has also received clearance in Switzerland by the Ethikkommission Nord-west und Zentralschweiz (Req-2019-00348).

Patient and public involvement

The research was done without public involvement.

RESULTS

The results section presents key findings elucidating the characteristics of resilience that have been salient in Myanmar's response to Cyclone Nargis. The results are categorised into assets, barriers, and future opportunities for achieving health system resilience.

For peer review only - http://bmjopen.bmj.com/site/about/guidelines.xhtml

Foundational assets that can unlock Myanmar's health system resilience

Strong social capital comprised of informal channels and trusted networks

Most participants pointed to the extensive role that the informal sector plays in filling the gaps of the health system. A case in point, the ambulance system in Myanmar is currently charity-based and subjected to resource availability from various donors. The township hospitals themselves have to seek out appropriate charity groups to maintain the referral system comprised of ambulances and drivers. Likewise, when Cyclone Nargis hit, there were various community-based organisations as first responders of the crisis, offering food, shelter and basic commodities to the affected villages. Strong social capital in Myanmar were exhibited mainly through informal channels, whether it be via monasteries and community-based charity groups. They were able to operate without legal mandates and special memorandum of understanding, precisely because they have obtained people's trust and leveraged the familiarity of their own network. One NGO worker described:

> *"There was an existing social community that was ready to donate to religious events and they were actively involved and participated in the relief efforts after Nargis hit. Each village has a social committee and the religious leaders in the monastry have their own shelters for the affected people who sought refuge. The communities themselves know their needs and gaps the best, which can build resilience." (I09)*

In a context where access to the affected regions were limited for the first three weeks after the cyclone, community engagement proved vital in offering timely support. It was apparent that participants who visited the affected areas experienced severe logistical challenges. Some of the areas were only accessible by boat or in off-road conditions where a robust 4x4 vehicle was required. In this endemically challenging environment, mobile clinics were established to reach the communities in remote areas. All the more in these contexts, community-based resilience was not optional but imperative.

> *"People had to travel long distances to access health care, which severely limited preventative care and people accessing the health system…One thing that consistently stood out to me was the incredible self-sufficiency of the communities themselves, their resilience, and their ability to get back on their feet with the limited resources that they had." (I11)*

Referring back to the example of charity-based ambulances, a few participants stated their concerns that this heavy reliance on informal channels can in turn be a barrier towards building basic systematic preparedness, as often these groups can run without standardized procedures and beget further inequalities in service delivery.

> *A basic preparedness however is not equipped because of this reliance on informal channels. Not all township hospitals have ambulances. Apparently, there is no standard. An ambulance and a driver are not part of a township health department set up. (I06)*

As evidenced through Cyclone Nargis, social capital albeit informal in nature served as an asset for Myanmar in both crisis and calm.

Cultural and religious commitment as a propeller for motivation

Underpinning the strong social capital lay the commitment of community-based groups as the backbone of relief efforts. Many attested that Myanmar's history of prolonged isolation and under-development led to a stronger demonstration of resilience at the community level especially in a situation of emergency. In Myanmar, volunteerism is a common method of civic participation (29). The motivation for this commitment appeared to have stemmed from the people's religious and cultural beliefs to give to charity and donate to the less fortunate, which also manifested in the case of Nargis. A UN staff member shared:

For peer review only - http://bmjopen.bmj.com/site/about/guidelines.xhtml

"People in a community supporting other people in need is a powerful response. Most believe that it's because they were closed off for so long, and it was just helping themselves, and the nature of the general beliefs and teachings of the country. People will go to churches, pagodas, temples, monastries or other public buildings to shelter, and the community there will provide food for a couple of days generally." (I07)

Traditional social welfare support groups revolving often through informal channels played a vital role at the village level and many self-help groups sprung up spontaneously to join the momentum (30). The monasteries even had an informal system to store food collected through donations and shared them with the communities in need. They were the sole sources of aid in the immediate aftermath of Cyclone Nargis until support arrived from the government and UN agencies, which proved as a clear asset. Several interviewers echoed that these social determinants of religion and culture provided a supporting environment for subsequent relief projects to flourish.

"The Buddhist culture compels people to donate to those in need and the monks. The first responses have always come from the people/monasteries, later from the government and finally from the UN agencies. It's because it's part of the culture, all these donations." (I01)

"There are social determinants at play in the response to Nargis. At the time of the disaster, 90% of the people were Buddhist. Those from non-affected townships even came. Came to donate. This is the culture of Buddhism. Supporting environment was religion." (I05)

Community resilience in Myanmar seems to have been built on a stronger foundation because it stems from religion and culture as a motivating force. The powerful source of solidarity within some of the affected communities weathered through generations of political and ethnic divisions.

Barriers to Myanmar's journey towards resilience

Historical legacy affecting transparency, openness and decentralisation

When reflecting upon the aftermath of the cyclone, all participants called out the lack of transparency and openness in how the government had dealt with the crisis at hand. The government at the time did not convey the exact situation analysis to the public, withheld key information about the cyclone, and controlled data such as the degree of damage and casualties incurred. Such lack of transparency not only undermined public trust towards the government but also limited international response teams from accessing Myanmar, which resulted in an overall delay in collective response efforts.

"The government at that time was so quiet and the civil society has not seen the first response from the government. INGOs were not able to access the area until three weeks after the cyclone hit. Perhaps, this is the reason why the government also tried to hide the information as much as possible, for instance, the number of casualties, number of shelters required, etc. We learnt the lack of government information is tied to the lack of accountability." (I10)

Though the reasons may be complex, most respondents pointed to Myanmar's long history of military rule that has operated based on a rigid hierarchy and centralized decision-making system, a context that may not have been conducive for disaster management. There was no proper disaster management mechanism in place; rather, coordination was led through the health cluster. As it had been a highly centralized system, however, the senior general still had the final say and was initially not open to collaborating with the outside world.

"Everything had to be reported and decided by the senior general (Than Swe)..Whatever he said, everybody has to do it." (I04)

To this day, the Memorandum of Understanding (MoU) is signed at the national level, not with the state. The requests may be made locally, but the final say comes centrally. One participant stated that this hierarchical structure produces dissonant voices at different levels, complicating the decision-making process and posing a risk of not meeting the actual needs on the ground. Another participant echoed this sentiment:

> *"Myanmar is too centralized, which poses a key challenge towards building a resilient health system. Evidence-based planning at the local level is not possible. For example, prevention of the polio outbreak that occurred in Hpa-bun would have been possible if local collaboration with the ethnic organisation could be done at the state level, where states have the decision making power and have fiscal decentralisation." (I06)*

Many participants pointed to theadded layer of complication from the presence of ethnic armed groups in self-administered zones that are run independently of the government as a result of its ongoing strife towards independence (31). These special subnational administrations run by multiple ethnic groups not only have their own health system parallel to that of the Myanmar government but selectively collaborates with local NGOs and community-based groups where there are no conflicts of interest. One participant shared that the polio outbreak in May of 2019 in one of these self-administered regions was particularly difficult to contain due to lack of information shared and restricted access to the area.

> *"Often ethnic health organisations work in these areas to provide services, and some are in fact coordinating well with the ministry. But, access can be difficult, as in the case of the polio outbreak, and the ethnic health organisation wouldn't let the ministry in nor UN agencies. They only allow one NGO they trust to have access. "If you're looking at some kind of outbreak, there is a needed preparedness element by the ministry, but they also need to consider this other factor that is not something that other countries should necessarily worry about." (I06)*

Often UN agencies and NGOs offer to bridge these gaps in services, yet these two parallel systems pose great challenges for international actors to maneuver through the bureaucracies in these conflict zones. It is an ongoing challenge that Myanmar needs to overcome in order to reach an inclusive state of resilience at the national level.

Lack of investments in long-term variables of resilience

Cyclone Nargis revealed the fragility of its health system, especially the areas that had required long-term investments. One participant that had worked with the UN agency during this time depicted how dire the situation had been.

> *"The basic fabrics of the health system were not existing. There was not a place to even fit supplies, medicines and computers. Many RHCs were not functioning well, many times lacking midwives and essential medicine. The nurse to patient ratio was too low and building the nursing infrastructure was critical." (I04)*

According to these first-hand accounts, Myanmar still had a very basic infrastructure, township hospitals covering up to 200,000 people, and a rural health centre serving around 20,000-25,000 people (14). A typical rural health centre had a midwife but this was not necessarily the case in remote and hard-to-reach villages. Health worker shortages came up in all the interviews as a major obstacle for Myanmar's achieving its Universal Health Coverage goal, let alone its journey towards resilience. One UN staff indicated that on average only 54% of the sanctioned health worker posts are filled in Myanmar and building a strong human resource cadre that is evenly distributed across the health system requires a conscious long-term commitment and investment from the government.

> *"Though development cooperation can support buildings, supplies and equipments, robust human resources require a long-term commitment and investment that should come from the government..I*

For peer review only - http://bmjopen.bmj.com/site/about/guidelines.xhtml

mean, the largest issue of Myanmar is that you have only 54% of sanctioned posts filled. Even the 54%, if you see differences in regions." (I06)

All participants stressed the importance of investing in long-term infrastructure, including its health workforce across the country, particularly focusing on extending its health system coverage to remote and vulnerable areas in order to build resilience of the health system as a whole. However, the stark inequalities in remote areas and the frequent staff turnover at all levels of the government were reflections of poor working conditions and weak human resource policies. Participants currently working for a major UN agency shared that funding additional trainings may not be the solution to this health workforce challenge.

"Because of the weakness in the human resource policy, and the working nature of the government job, many government staff are resigning, quitting their jobs. We are losing qualified candidates. Human resource is a major challenge...I know many trainings were supported by organisations and donors, but the problem is the retention of human resource. After the trainings, they move or resign. I think training should not be the key weakness." (I02)

Some specifically referred to the importance of foresight for these long-term commitments. There seems to be a significant gap between plans on paper and implementation as mentioned by one civil society representative:

"The challenge in the state/region is human resource shortages, especially medical doctors. The analysis of the ministry of health national health plan reveals that across the country there is a health service gap due to the lack of human resources. There are not enough medical doctors trained through the medical universities. The government already has a plan, but there is a big implementation gap." (I10)

Myanmar's future for resilience

Harnessing social capital to unleash the power of collective civic duty through civil society groups

As echoed by multiple participants, Myanmar appears to have the ingredients for resilience within its own respective communities. Not only did existing community-based groups act as first responders of Cyclone Nargis albeit decades of dictatorship, but even the coordination took place naturally and provided mutual support. It was simply the people exhibiting civic duty and playing their due role in the society. One representative of a local civil society group shared that health system resilience may be reached when civil society groups build community resilience and start working together with a health system that is also investing in building its strengths.

"At the time of the cyclone, the local civil society groups in each area organised and coordinated with each other by themselves. We haven't seen any government, like red cross, or police. We haven't seen any solider. During the Cyclone Nargis, we have seen the communities, the people, how did they respond. They came out to the street and they supported each other. They tried to remove all the trees and blocks. We learnt from them. We have strong resilient communities. Communities know best what the biggest challenge and needs are in those areas. If we work together, community resilience strengthening and health system strengthening, we could be working towards resilience." (I10)

The informal channels of self-formed community-based groups can be a strong basis for the institutionalization of civil society groups that have a rather complicated relationship with the government. Since Myanmar has opened up in 2011 under President Thein Sein, its reform process has gradually enabled a platform for civil society to establish civil liberties through its '2008 Constitution,' with the article 354 stating the right to freedom of expression, assembly and association (32). Furthermore, the Parliament has enacted the Association Registration Law, which defines and clarifies local and international non-governmental organizations and stipulates requirements to register with the government. These reforms have gradually shifted the landscape of civil society engagements at all levels of the country.

For peer review only - http://bmjopen.bmj.com/site/about/guidelines.xhtml

Even at the national level, the government has been more receptive to the inputs of the civil society groups, including the Ministry of Health and Sports with many of its community-driven development projects. There are certainly growing opportunities for civil society groups to participate in the various development sector working groups (33). One participant reflects on the evolution of civil society engagement since Cyclone Nargis and projects that no crisis can be handled without the involvement of the country's civil society groups.

> *"The health system at that time was already weak and the ministry of health didn't have a strong coordination with the civil society or community groups. The government organised many meetings but had no idea how to work with the local civil society. Only in the last four years did this relationship change. The minister of health stressed the importance of working with CSOs across the country. If an outbreak were to happen, the ministry of health cannot handle the situation without civil societies." (I10)*

Connecting its overarching health goals with long-term investments to improve resilience

As Myanmar is situated in a disaster-prone area, it has naturally developed a local expertise over time through handling its yearly floods. One participant stated that there is an emergency fund stipulated by law to set aside a disaster management fund that can be used to support sub-national levels in case of a potential crisis. How the legal frameworks and plans align with its budgeting and allocation, however, is a different issue.

A recurring theme that transpired when discussing the government's response to the cyclone and other subsequent crises were the emergence of discrepancies between planning, budgeting and implementation. As there has been no clear alignment between its long-term health policy and crisis management, the targeted investment towards building system-level resilience has also lagged behind.

> *"There is very remote connection between planning and budgeting. It happens both at the national and local levels. Preparedness is there on paper." (I06)*

One participant called this the ability to "expand and contract," which also corresponds to the concept of adaptiveness in Kruk et al's framework. It is exhibited by the ability to reallocate its resources to where it is most needed on time and to delegate the authority to the appropriate decentralized administration when necessary (6). It is a vital characteristic of resilience, which is a byproduct of long-term investments and painstaking reforms (34).

> *"This kind of planning, the constant expansion and contraction that you would need in an environment like Myanmar that's so natural disaster heavy, is difficult." (I07)*

Many participants agreed that Myanmar ought to invest towards building long-term infrastructure especially in remote areas and prioritize in its human resource preparedness in order to build resilience in the system for future shocks. This may involve employing creative ways to increase staff retention through the engagement of the private and non-profit sectors. More importantly, however, these plans ought to connect back to its overarching national goal for Universal Health Coverage and its momentum to strengthen its commitment towards International Health Regulations.

> *"In order to improve health system resilience, it is necessary to improve infrastructure needed at the village level… A challenge in building the long-term capacity of health workers, however, is the frequent turnover of staff. Mostly staff assigned for the rural health centres are based there for 1-2 years and move to a better place. Turnover was quite quick." (I09)*

DISCUSSION

For peer review only - http://bmjopen.bmj.com/site/about/guidelines.xhtml

This study explored the degree to which key characteristics of health system resilience manifested in Myanmar's response to Cyclone Nargis and their respective historical and socio-political determinants. As health systems are complex and evolving in nature, it is important to predicate this study on the view that resilience is a capacity that can be nurtured with time and commitment (35). Moreover, facets of resilience should not be seen as disconnected inputs, but rather a holistic result of targeted investments and a series of structural reforms (10), (34).

In the case of Myanmar, it had been the 'everyday resilience' of its frontline workers and communities that had been built through its day-to-day chronic struggles in the context of its weak governance and political and economic vulnerabilities, that in turn prepared them for the unexpected shock (8). As depicted in Figure 2, key themes that emerged from the analysis have been organised into assets, barriers and future opportunities in describing Myanmar's journey towards resilience.

First, social capital and motivation have surfaced as key foundational assets in Myanmar that are indicative of its community resilience. The power of collective civic duty filled the service gaps that the government had failed to provide in the immediate aftermath of the cyclone. The social capital present in Myanmar during Cyclone Nargis were comprised of informal channels and trusted networks in the communities, its motivation stemming from a deeper faith rooted in religion and culture. A recent qualitative synthesis highlighted social capital as the interconnecting mechanism common to both health system strengthening and resilient responses to crises, a powerful tool that can unlock resilience of the health system (10). This study extends the notion a step further by demonstrating that the power of social capital can go beyond its health system capacity. Social capital may in fact play a more important role in providing relief from the cyclone, just as it had been reported as being more vital in mitigating the spread of the Ebola virus than any donor-driven short-term interventions (13). The pivotal role social capital plays in building community resilience have been well documented by other studies in the context of both natural and man-made disasters (36), (37), (38), (39), (40). When communities themselves start carrying out critical health system functions, this collective momentum for community resilience prompts what Barker et al calls a 'fortuitous cycle' of increased trust, improved communication and continued meaningful community engagement, all of which are crucial preconditions for enabling health system resilience (11).

Second, Myanmar's historical legacy of prolonged military rule and a lack of long-term investments posed as major barriers to achieving resilience. The lack of transparency and openness stood out as the biggest obstacle towards crisis management, which undermined public trust towards the government and hampered international response teams from accessing Myanmar in the initial stages. Myanmar is a prime example of a post-colonial state which development experts describes as a 'conflict trap' (41). The legacy of the colonial experience, ethnic insurgencies, and a successive military rule, shifted all of its government's priorities to regaining its national sovereignty and ensuring security through increased military spending (42). Consequently, little had been left for health and social welfare, with only approximately 2% of its GDP spent on health care shortly before the cyclone hit (30). It is no surprise that Myanmar's health system was fragile and not ready to handle a disaster of such magnitude.

Governance practices influence organizational resilience and decentralized governments are certainly at an advantage to exhibit the necessary flexibility and timely responses required in crisis management (2). Having a decentralized governance structure itself, however, does not guarantee a successful response. China had no national disaster management agency nor related policies during the Sichuan earthquake, but effectively controlled the emergency with

For peer review only - http://bmjopen.bmj.com/site/about/guidelines.xhtml

its President's charismatic leadership and vertical administrative structure (43). Myanmar's centralized and hierarchical system could have been used for Myanmar's advantage, but an adequate disaster management had not been the priority of the government.

A common pattern across failed attempts in responding to a crisis was to prioritize on the 'fast variables,' delivering services in the form of temporary shelters and supplies without considering its longer-term infrastructure building, as it had been in the case of Liberia during its Ebola crisis (44). Building resilience, however, is much more than preparedness, as Kruk et al states, that it requires investing in systems and its corresponding 'slow variables' that can function in both crisis and calm (6). A health system that has a clear foresight for its people will strengthen its health system infrastructure and train its human resource cadre to build the foundations of its health system (10). In the context of Myanmar, it would mean to apply a systems thinking approach in examining its past shortcomings and redesigning its institutions and organisational structures to serve its evolving purpose and prepare for future shocks (45).

Third, Myanmar may channel its assets into opportunities by using its growing civil society platforms and leveraging its long-term national health goals. Cyclone Nargis revealed that Myanmar has the key ingredients for resilience within its own communities through its people exhibiting their civic duty. Civil society has had a long history in Myanmar dating back to its informal Buddhist associations in the traditional kingdoms and various other religious groups organizing social and welfare programmes during its colonial period and beyond (46). Since Cyclone Nargis, the space for civil societies further expanded through its legal reforms but at the end of the day only a few have been able to obtain official registration and secure a sustainable funding mechanism (46). According to the Civil Society Survey conducted in 2019, despite its marked progress, a wide range of obstacles still remain and hinder civil societies from reaching their goals (32). Myanmar has set ambitious national goals to reach Universal Health Coverage (UHC) and to improve compliance to the International Health Regulations (IHR) in order to ensure healthcare and health security for all (22), (25). As evident in its neighbouring countries, however, civil society engagement and community feedback loops for policy and decision-making processes have shown as prerequisites to accelerate progress towards achieving these compendium of goals (47). Trust and accountability at all levels in the system are what builds the 'capacity of legitimacy,' a crucial component of resilient health systems, that can only be strengthened through an inclusive consultation process of engaging communities and civil society groups (7). Myanmar has to realize that strategically leveraging these opportunities may be the fastest route to building resilience.

For peer review only

The findings of this study offer tangible suggestions for Myanmar to invest on building its hard and soft long-term infrastructures in remote areas, prioritising in human resource preparedness, and redesigning institutions and organisational structures to serve its evolving purposes and to prepare for future shocks.

Study Strengths and Limitations

This study is empirical in nature, building on a health system resilience conceptual framework developed from a previous systematic review. The framework has been tested for the first time for its applicability in a lower-middle-income country. Regarding the study design, the long time lag between the actual event and data collection introduces a potential recall bias, which was partially offset by triangulation through supplementary data such as reports, event pictures and media clips provided by the participants. The topic of health system resilience has a potential to make a timely contribution to the health systems and policy research community, with lessons generated that can be transferrable to other contexts. Due to Covid-

For peer review only - http://bmjopen.bmj.com/site/about/guidelines.xhtml

19 pandemic's travel restrictions imposed during the data collection period, the study only represents the view of international organisations and non-governmental organisations, restricting the scope of the study to the perceptions of a single stakeholder group. Further studies should explore capturing a wider range of stakeholders, the perceptions of communities, frontline workers and government staff that may be able to offer a more holistic view of the phenomenon.

CONCLUSION

This study revealed that resilience in the health system can be built through targeted investments towards building the foundations of resilience to better prepare for future shocks. In the case of Myanmar, social capital and motivation, which surfaced as its foundational assets, may be channelled into opportunities that can help achieve its long-term health goals, accelerating its journey towards resilience in the health system.

ACKNOWLEDGMENTS

The authors would like to extend appreciations to all the interviewees who carved out time to part take in this study. Special thanks to Dr. Nilar Tin for her valuable input on the initial study concept and for suggesting relevant interviewees.

CONTRIBUTORS

PYG and KW conceptualized the study; PYG collected and analysed the data with substantive input from SM and KW. PYG developed the first draft of the manuscript and all authors contributed to the subsequent and final drafts.

FUNDING

This article is part of the PhD project that has received funding from the European Union's Horizon 2020 research and innovation programme under the Marie Skłodowska-Curie grant agreement No. 801076, through the SSPH+ *Global PhD Fellowship Programme in Public Health Sciences (GlobalP3HS) of the Swiss School of Public Health*. The corresponding author is also a recipient of the Swiss Government Excellence Scholarship (ESKAS), provided by the Swiss Federal Commission for Scholarships for Foreign Students (FCS) with the award number 2018.0217. The funding bodies did not play a role in the design, analysis, and writing of the manuscript.

COMPETING INTERESTS

None declared

PATIENT CONSENT FOR PUBLICATION

Not required

ETHICS APPROVAL

The proposal for this study was approved in Myanmar by the Institutional Review Board of the University of Public Health and the Ministry of Health and Sports of the Republic of the Union of Myanmar (2020/Research/4). It has also received clearance in Switzerland by the Ethikkommission Nord-west und Zentralschweiz (Req-2019-00348).

DATA AVAILABILITY STATEMENT

For peer review only

For peer review only - http://bmjopen.bmj.com/site/about/guidelines.xhtml

This is a qualitative study of a small number of interviewees representing international organizations and NGOs. All data relevant to the study are included in the article or as supplementary information. Making the full data set publicly available could potentially breach the privacy commitment made to the participants upon obtaining informed consent, as well as the ethics approval granted by both the Institutional Review Board of the University of Public Health in Myanmar and Ethikkommission Nord-west und Zentralschweiz in Switzerland. Therefore, the authors will not make the full transcripts available to a wider audience.

Figure 1. Grimm et al.'s health system resilience framework (10)

Figure 2. Assets, barriers and opportunities for Myanmar's journey towards health system resilience

REFERENCES

1. Global HS. Vancouver statement for the fourth global symposium on health systems research. 2016.
2. Barasa E, Mbau R, Gilson L. What Is Resilience and How Can It Be Nurtured? A Systematic Review of Empirical Literature on Organizational Resilience. International Journal of Health Policy and Management. 2018;7(6):491-503.
3. Fridell M, Edwin S, Von Schreeb J, Saulnier DD. Health system resilience: what are we talking about? A scoping review mapping characteristics and keywords. International Journal of Health Policy and Management. 2020;9(1):6.
4. Biddle L, Wahedi K, Bozorgmehr K. Health system resilience: a literature review of empirical research. Health Policy Plan. 2020;35(8).
5. Kruk ME, Myers M, Varpilah ST, Dahn BT. What is a resilient health system? Lessons from Ebola. The Lancet. 2015;385(9980):1910-2.
6. Kruk ME, Ling EJ, Bitton A, Cammett M, Cavanaugh K, Chopra M, et al. Building resilient health systems: a proposal for a resilience index. BMJ. 2017:j2323.
7. Blanchet K, Nam SL, Ramalingam B, Pozo-Martin F. Governance and Capacity to Manage Resilience of Health Systems: Towards a New Conceptual Framework. Int J Health Policy Manag. 2017;6(8):431-5.
8. Gilson L, Barasa E, Nxumalo N, Cleary S, Goudge J, Molyneux S, et al. Everyday resilience in district health systems: Emerging insights from the front lines in Kenya and South Africa. BMJ Global Health. 2017;2.
9. Barasa EW, Cloete K, Gilson L. From bouncing back, to nurturing emergence: reframing the concept of resilience in health systems strengthening. Health Policy Plan. 2017;32:91-4.
10. Grimm PY, Oliver S, Merten S, Han WW, Wyss K. Enhancing the Understanding of Resilience in Health Systems of Low- and Middle-income Countries: A Qualitative Evidence Synthesis International Journal of Health Policy and Management. 2021:-.
11. Barker KM, Ling EJ, Fallah M, VanDeBogert B, Kodl Y, Macauley RJ, et al. Community engagement for health system resilience: evidence from Liberia's Ebola epidemic. 2020;35(4):416-23.
12. Bhandari S, Alonge OJHrp, systems. Measuring the resilience of health systems in low-and middle-income countries: a focus on community resilience. 2020;18(1):1-19.
13. Alonge O, Sonkarlay S, Gwaikolo W, Fahim C, Cooper J, Peters DJGha. Understanding the role of community resilience in addressing the Ebola virus disease epidemic in Liberia: a qualitative study (community resilience in Liberia). 2019;12(1):1662682.
14. Group TC. Post-Nargis Joint Assessment. 2008.
15. EM-DAT: The Emergency Events Database [Internet]. Universite catholique de Louvain (UCL). Available from: www.emdat.be.

For peer review only

For peer review only - http://bmjopen.bmj.com/site/about/guidelines.xhtml

16. Warr P, Aung LLJWD. Poverty and inequality impact of a natural disaster: Myanmar's 2008 cyclone Nargis. 2019;122:446-61.
17. Stover E, Vinck PJJ. Cyclone Nargis and the politics of relief and reconstruction aid in Burma (Myanmar). 2008;300(6):729-31.
18. Secretary-General's press conference at Hotel Sedona in Yangon [press release]. 23 May 2008.
19. David Eckstein VK, Laura Schäfer, Maik Winges. Global climate risk index 2020 Germanwatch; 2019.
20. INFORM Global Risk Index 2020 [Internet]. European Commission 2019. Available from: https://drmkc.jrc.ec.europa.eu/inform-index/.
21. UNOCHA. HCT Myanmar- Inter-agency emergency response preparedness (ERP) plan 2017.
22. Ministry of H, Sports tRotUoM. Myanmar National Health Plan 2017-2021. 2016.
23. Pyone T, Aung TT, Endericks T, Myint NW, Inamdar L, Collins S, et al. Health system governance in strengthening International Health Regulations (IHR) compliance in Myanmar. 2020;5(11):e003566.
24. Joint external evaluation of IHR core capacities of The Republic of the Union of Myanmar: executive summary. . World Health Organization; 2017
25. National Action Plan for Health Security (NAPHS) 2018-2022. Ministry of Health and Sports, the Republic of the Union of Myanmar 2018.
26. Naderifar M, Goli H, Ghaljaie FJSiDoME. Snowball sampling: A purposeful method of sampling in qualitative research. 2017;14(3):1-6.
27. Guest G, Bunce A, Johnson L. How Many Interviews Are Enough?: An Experiment with Data Saturation and Variability. Field Methods. 2006;18(1):59-82.
28. Gale NK, Heath G, Cameron E, Rashid S, Redwood S. Using the framework method for the analysis of qualitative data in multi-disciplinary health research. BMC Medical Research Methodology. 2013;13(1).
29. Dove CJAJoS-EAS. Giving Trends in Myanmar: More Than Merit Making. 2017;10(2):205-22.
30. The Republic of the Union of Myanmar Health System Review: World Health Organization; 2015 2015.
31. Jolliffe KJTAF. Ethnic armed conflict and territorial administration in Myanmar. 2015:89.
32. Buschmann A, Soe AJAaS. Authoritarian Impediments to Civil Society in Contemporary Myanmar: Findings from the Myanmar Civil Society Survey 2019. 2020.
33. Civil society briefs: Myanmar. Asian Development Bank 2015.
34. Kutzin J, Sparkes SPJBotWHO. Health systems strengthening, universal health coverage, health security and resilience. 2016;94(1):2.
35. Topp SM. Power and politics: the case for linking resilience to health system governance. BMJ Specialist Journals; 2020.
36. Bianca RJTRoAS. Influence of social capital on community resilience in the case of emergency situations in Romania. 2018;14(54):73-89.
37. Aldrich DP, Meyer MA. Social Capital and Community Resilience. American Behavioral Scientist. 2015;59:254-69.
38. Pfefferbaum B, Van Horn RL, Pfefferbaum RLJCSWJ. A conceptual framework to enhance community resilience using social capital. 2017;45(2):102-10.
39. Aldrich DP. The importance of social capital in building community resilience. Rethinking resilience, adaptation and transformation in a time of change: Springer; 2017. p. 357-64.
40. Grimm PY, Saulnier DD. Civic Duty: A Booster for Resilience? International Journal of Public Health. 2021;66.
41. Smith M. State of strife: The dynamics of ethnic conflict in Burma: Institute of Southeast Asian Studies; 2007.
42. Than TMM. Myanmar: preoccupation with regime survival, national unity, and stability. Standford: Stanford University Press; 1998. 390-416 p.

For peer review only - http://bmjopen.bmj.com/site/about/guidelines.xhtml

43. Lin L, Ashkenazi I, Dorn BC, Savoia E. The public health system response to the 2008 Sichuan province earthquake: a literature review and interviews. Disasters. 2014;38(4):753-73.

44. Ling EJ, Larson E, Macauley RJ, Kodl Y, VanDeBogert B, Baawo S, et al. Beyond the crisis: did the Ebola epidemic improve resilience of Liberia's health system? Health Policy Plan. 2017;32(suppl_3):iii40-iii7.

45. Arnold RD, Wade JPJPCS. A definition of systems thinking: A systems approach. 2015;44(2015):669-78.

46. Kramer T. Civil society gaining ground: Opportunities for change and development in Burma: Transnational Institute Amsterdam; 2011.

47. Yeoh E-K, Johnston C, Chau PYK, Kiang N, Tin P, Tang JJHS, et al. Governance functions to accelerate progress toward universal health coverage (UHC) in the Asia-Pacific Region. 2019;5(1):48-58.

For peer review only

For peer review only - http://bmjopen.bmj.com/site/about/guidelines.xhtml

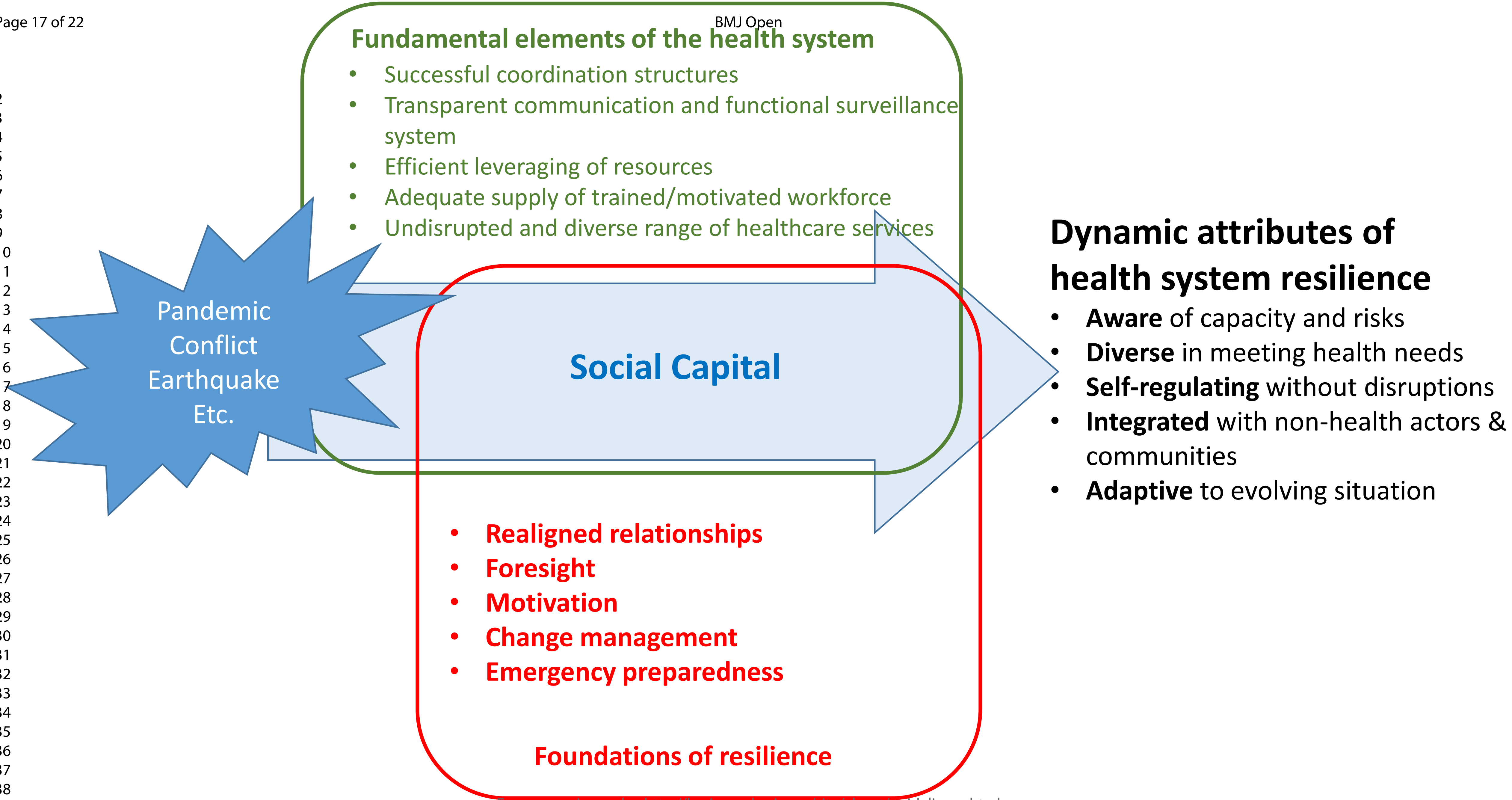
Fundamental elements of the health system
• Successful coordination structures
• Transparent communication and functional surveillance system
• Efficient leveraging of resources
• Adequate supply of trained/motivated workforce
• Undisrupted and diverse range of healthcare services
Pandemic
Conflict
Earthquake
Etc.
Social Capital
• Realigned relationships
• Foresight
• Motivation
• Change management
• Emergency preparedness
Foundations of resilience
Dynamic attributes of health system resilience
• Aware of capacity and risks
• Diverse in meeting health needs
• Self-regulating without disruptions
• Integrated with non-health actors & communities
• Adaptive to evolving situation

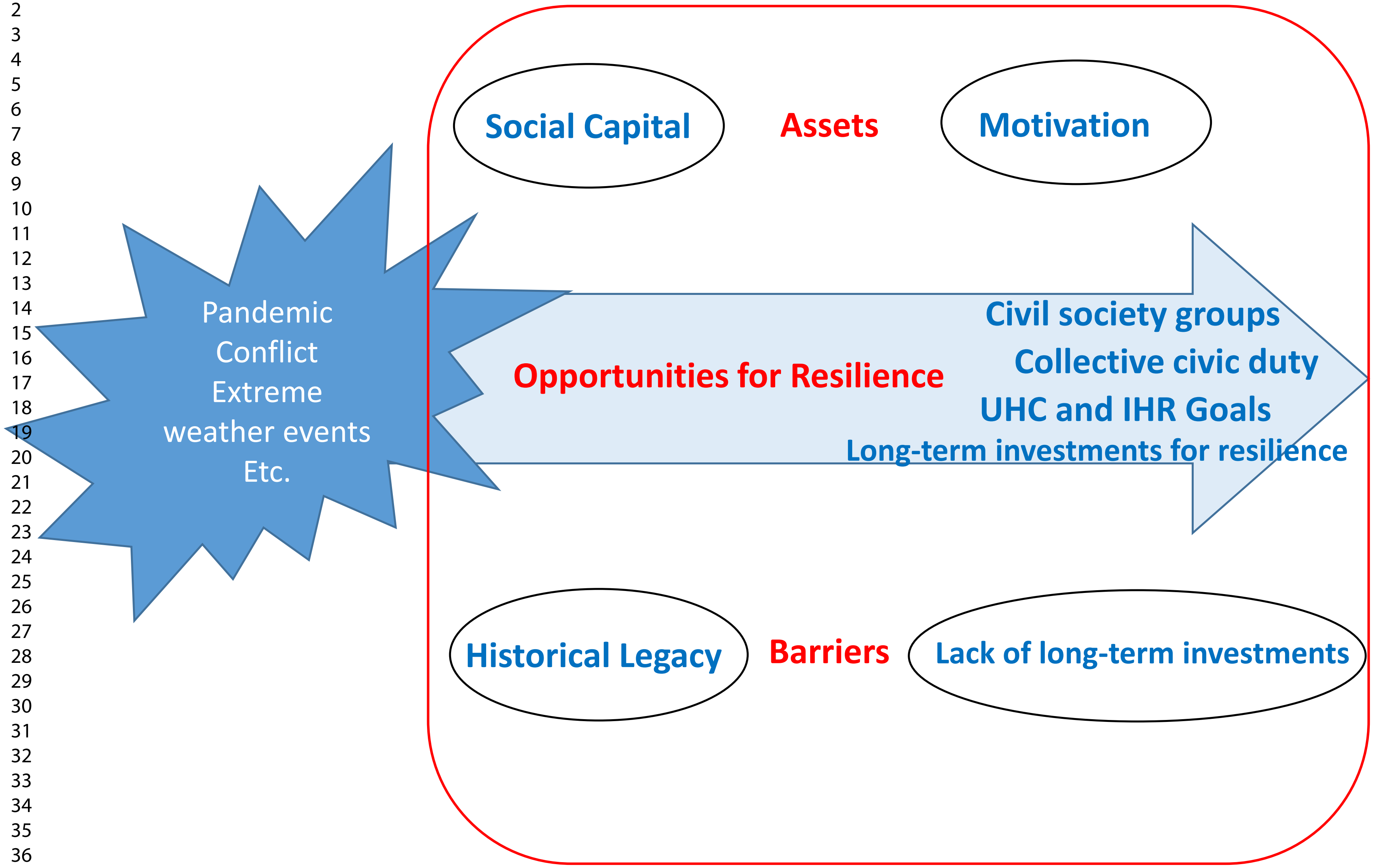

Health system resilience

- **Aware** of capacity and risks
- **Diverse** in meeting health needs
- **Self-regulating** without disruptions
- **Integrated** with non-health actors & communities
- **Adaptive** to evolving situation

(Kruk et al. 2015, 2017)

SUPPLEMENTARY FILE. INTERVIEW GUIDE

Study Title: Health System Resilience: key for unlocking the complex health system strengthening agenda

Investigator: ________________________ **Phone number:**____________________________

Organisation/Department: ______________________________ **Date: |__|__|__|__|__|__|**

Position/Title: ____

Stakeholder Group:

□ Central Government

□ Local Government (Township, local health committees, member of parliament, etc)

□ Health workers

□ Int'l Agencies (UN, NGOs, Bilateral, etc)

Number of participants: |__|__|

DRAFT SEMI-STRUCTURED IN-DEPTH INTERVIEW QUESTIONS

Introduction: purpose of research, use of audio tape recorder, confidentiality

I. PARTICIPANT INTRODUCTION

1. What is your role when the country is hit with an external shock, whether it be a natural disaster, unforeseen pandemic, etc.

   Prompt: examples of disaster management, pandemic, natural disasters

II. CASE 1: CYCLONE NARGIS (2008)

2. (General) Can you please describe in detail the account of Cyclone Nargis?

   Probe: What was your title/position at the time?

   What was the response protocol (written or perceived norm)?

   What actions (out of the norm) did you take?

   How did you respond as an individual?

3. (General) Can you please describe in detail the response actions taken as an organisation that you were a part of at the time?

   Probe: Decision-making protocol? Evidence?

For peer review only

4. (General) Can you comment on one strength and weakness that the health system exhibited during Cyclone Nargis?

Probe: Which key element was present or missing during the incident?

5. (Dimension 1_Aware) At the time of Cyclone Nargis, how was the health system monitoring and tracking its health risks?

6. (Dimension 1_Aware) Once the disaster hit, who was included in the communication channel?

7. (Dimension 1_Aware) Who made the decisions regarding resource allocation and coordination efforts?

8. (Dimension 2_Diverse) At the time of Cyclone Nargis, were a range of health services still offered in health facilities (business as usual) or was the quality/quantity of services compromised? Why?

9. (Dimension 3_Self-regulating) Can you recall the extent of non-state organisations intervening to provide support at the time of crisis? (e.g. NGOs, Int'l Orgnisations, private actors, etc)

10. (Dimension 3_Self-regulating) At national level, were there reserve capacity (budget, HR, other resources) to summon quickly?

11. (Dimension 4_Integrated) Can you comment on the level of coordination between government, global and private actors at the time of the crisis?

12. (Dimension 4_Integrated) Who was the main actor responsible for coordination?

13. (Dimension 5_Adaptive) Was there at the time flexibility when it came to spending budget to respond to the emergency?

14. (Dimension 5_Adaptive) Can you comment on how much decision-making was possible at the decentralised/local level?

15. What was the main lessons learnt after undergoing Cyclone Nargis?

III. CONCEPT OF HEALTH SYSTEM RESILIENCE (HSR) AND GENERAL QUESTIONS

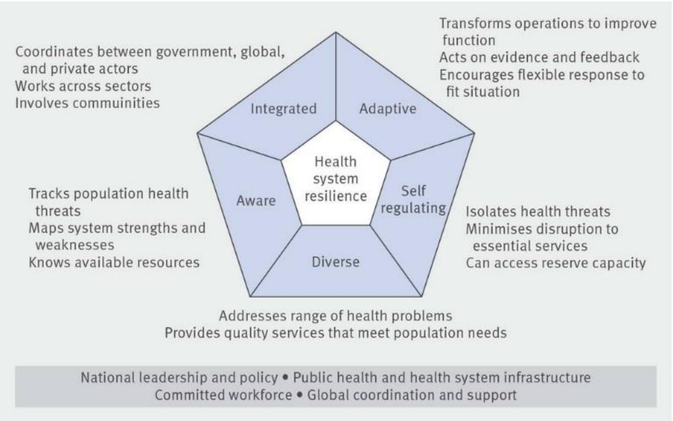

Figure 1. Resilient Health System Framework

16. Reviewing the framework above, how would you describe Myanmar's health system resilience generally?

Probe: Can you give concrete examples that indicate Myanmar's HSR?

17. What is Myanmar's strength/weakness when it comes to resilience?

18. What do you think is the most important element of resilience?

IV. PREPAREDNESS AND FUTURE SHOCKS

19. What may be the biggest threat/risk for Myanmar?

20. At this point in time, how would you describe Myanmar's preparedness for future shocks?

21. What national policies are in place to enable the Ministry of Health to be resilient to future shocks?

22. Can you comment on the efforts taken by the Ministry of Health and Sports to improve preparedness? (HR allocation, crisis management team, emergency plan, trainings undertaken, protocols, etc)

23. What would you advise to improve health system resilience in Myanmar?

Thank you for your time and heartfelt responses to the interview questions.

For peer review only

Consolidated criteria for reporting qualitative studies (COREQ): 32-item checklist

Developed from:
Tong A, Sainsbury P, Craig J. Consolidated criteria for reporting qualitative research (COREQ): a 32-item checklist for interviews and focus groups. *International Journal for Quality in Health Care*. 2007. Volume 19, Number 6: pp. 349 – 357

No. Item	Guide questions/description	Reported on Page #
**Domain 1: Research team and reflexivity**		
Personal Characteristics		
1. Inter viewer/facilitator	Which author/s conducted the inter view or focus group?	pg.4
2. Credentials	What were the researcher's credentials? E.g. PhD, MD	pg.4
3. Occupation	What was their occupation at the time of the study?	pg.4
4. Gender	Was the researcher male or female?	N/A
5. Experience and training	What experience or training did the researcher have?	pg.4
Relationship with participants		
6. Relationship established	Was a relationship established prior to study commencement?	pg.4
7. Participant knowledge of the interviewer	What did the participants know about the researcher? e.g. personal goals, reasons for doing the research	pg.4
8. Interviewer characteristics	What characteristics were reported about the inter viewer/facilitator? e.g. Bias, assumptions, reasons and interests in the research topic	pg.4
**Domain 2: study design**		
Theoretical framework		
9. Methodological orientation and Theory	What methodological orientation was stated to underpin the study? e.g. grounded theory, discourse analysis, ethnography, phenomenology, content analysis	pg.4
Participant selection		
10. Sampling	How were participants selected? e.g. purposive, convenience, consecutive, snowball	pg.3
11. Method of approach	How were participants approached? e.g. face-to-face, telephone, mail, email	pg.4
12. Sample size	How many participants were in the study?	pg.3
13. Non-participation	How many people refused to participate or dropped out? Reasons?	N/A

For peer review only

Setting		
14. Setting of data collection	Where was the data collected? e.g. home, clinic, workplace	pg.4
15. Presence of non-participants	Was anyone else present besides the participants and researchers?	N/A
16. Description of sample	What are the important characteristics of the sample? e.g. demographic data, date	N/A
Data collection		
17. Interview guide	Were questions, prompts, guides provided by the authors? Was it pilot tested?	pg.4
18. Repeat interviews	Were repeat inter views carried out? If yes, how many?	N/A
19. Audio/visual recording	Did the research use audio or visual recording to collect the data?	pg.4
20. Field notes	Were field notes made during and/or after the inter view or focus group?	pg.4
21. Duration	What was the duration of the inter views or focus group?	pg.4
22. Data saturation	Was data saturation discussed?	pg.3
23. Transcripts returned	Were transcripts returned to participants for comment and/or correction?	pg.4
**Domain 3: analysis and findings**		
Data analysis		
24. Number of data coders	How many data coders coded the data?	pg.4
25. Description of the coding tree	Did authors provide a description of the coding tree?	N/A
26. Derivation of themes	Were themes identified in advance or derived from the data?	pg.4
27. Software	What software, if applicable, was used to manage the data?	MAXQDA 2018
28. Participant checking	Did participants provide feedback on the findings?	pg.4
Reporting		
29. Quotations presented	Were participant quotations presented to illustrate the themes/findings? Was each quotation identified? e.g. participant number	All throughout Results (pg. 4-9)
30. Data and findings consistent	Was there consistency between the data presented and the findings?	Yes (results/discussion)
31. Clarity of major themes	Were major themes clearly presented in the findings?	Results (pg.4-9)
32. Clarity of minor themes	Is there a description of diverse cases or discussion of minor themes?	pg.5

For peer review only
